# Individualized dynamic methylation-based analysis of cell-free DNA in postoperative monitoring of lung cancer

**DOI:** 10.1186/s12916-023-02954-z

**Published:** 2023-07-14

**Authors:** Kezhong Chen, Guannan Kang, Zhihong Zhang, Analyn Lizaso, Stephan Beck, Iben Lyskjær, Olga Chervova, Bingsi Li, Haifeng Shen, Chenyang Wang, Bing Li, Heng Zhao, Xi Li, Fan Yang, Nnennaya Kanu, Jun Wang

**Affiliations:** 1 Thoracic Oncology Institute and Department of Thoracic Surgery, Peking University People’s Hospital, Beijing, 100044 China; 2 Cancer Research UK Lung Cancer Centre of Excellence, University College London Cancer Institute, University College London, 72 Huntley St, London, WC1E 6DD UK; 3 Burning Rock Biotech, Guangzhou, 510300 China; 4 University College London Cancer Institute, University College London, 72 Huntley St, London, WC1E 6DD UK

**Keywords:** Lung cancer, Postoperative surveillance, Circulating tumor DNA (ctDNA), DNA methylation, Minimal residual disease (MRD)

## Abstract

**Background:**

The feasibility of DNA methylation-based assays in detecting minimal residual disease (MRD) and postoperative monitoring remains unestablished. We aim to investigate the dynamic characteristics of cancer-related methylation signals and the feasibility of methylation-based MRD detection in surgical lung cancer patients.

**Methods:**

Matched tumor, tumor-adjacent tissues, and longitudinal blood samples from a cohort (MEDAL) were analyzed by ultra-deep targeted sequencing and bisulfite sequencing. A tumor-informed methylation-based MRD (timMRD) was employed to evaluate the methylation status of each blood sample. Survival analysis was performed in the MEDAL cohort (*n* = 195) and validated in an independent cohort (DYNAMIC, *n* = 36).

**Results:**

Tumor-informed methylation status enabled an accurate recurrence risk assessment better than the tumor-naïve methylation approach. Baseline timMRD-scores were positively correlated with tumor burden, invasiveness, and the existence and abundance of somatic mutations. Patients with higher timMRD-scores at postoperative time-points demonstrated significantly shorter disease-free survival in the MEDAL cohort (HR: 3.08, 95% CI: 1.48–6.42; *P* = 0.002) and the independent DYNAMIC cohort (HR: 2.80, 95% CI: 0.96–8.20; *P* = 0.041). Multivariable regression analysis identified postoperative timMRD-score as an independent prognostic factor for lung cancer. Compared to tumor-informed somatic mutation status, timMRD-scores yielded better performance in identifying the relapsed patients during postoperative follow-up, including subgroups with lower tumor burden like stage I, and was more accurate among relapsed patients with baseline ctDNA-negative status. Comparing to the average lead time of ctDNA mutation, timMRD-score yielded a negative predictive value of 97.2% at 120 days prior to relapse.

**Conclusions:**

The dynamic methylation-based analysis of peripheral blood provides a promising strategy for postoperative cancer surveillance.

**Trial registration:**

This study (MEDAL, **ME**thylation based **D**ynamic **A**nalysis for **L**ung cancer) was registered on ClinicalTrials.gov on 08/05/2018 (NCT03634826). https://clinicaltrials.gov/ct2/show/NCT03634826.

**Supplementary Information:**

The online version contains supplementary material available at 10.1186/s12916-023-02954-z.

## Background

The detection of somatic mutations in circulating tumor DNA (ctDNA) had been shown to be a reflection of minimal residual disease (MRD) and help identify recurrence risk earlier than traditional strategies in patients with early-stage solid cancers, including lung cancer [1–3]. Despite the high specificity and positive predictive value (PPV) of ctDNA mutations, the extremely low ctDNA concentration in early-stage cancers poses a technological challenge. In addition, ctDNA-based mutation detection remains particularly challenging for patients with either low tumor burden or low tumor mutation burden, such as for patients with early-stage non-small-cell lung cancer (NSCLC) or *EGFR*-mutant lung adenocarcinoma [4]. Previous studies showed an improved sensitivity of patient-specific detection panels based on whole-exome sequencing (WES) of matched tissues; however, tumor-informed MRD based on WES are often costlyand inconvenient for clinical application [5, 6]. Sensitive and cost-effective assays detecting MRD remain to be explored.

DNA methylation is one of the most frequent epigenetic modifications and plays an important role in carcinogenesis and metastasis [7, 8]. Aberrant DNA methylation is more ubiquitous than somatic mutations, making it a sensitive biomarker for early cancer detection [9, 10]. A fixed DNA methylation sequencing panel coupled with in silico tumor-informed analysis could potentially achieve a satisfactory level of accuracy at a lower cost. Both global methylation status and tumor-specific methylation panels have previously been reported for early cancer detection [11–14] but the feasibility of methylation-based MRD detection and cancer surveillance remains relatively unexplored. So far, no study has elaborated on dynamic changes of cancer-related methylation in perioperative blood samples of early-stage cancers, which is critical for establishing a novel approach for methylation-based MRD detection.

This prospective observational study (MEDAL, **ME**thylation-based **D**ynamic **A**nalysis for **L**ung Cancer) investigated the dynamics of cancer-related methylation and the feasibility of methylation-based postoperative surveillance in patients with resected lung cancer [15]. We investigated the utility of an individualized tumor-informed methylation-based MRD (timMRD) model in evaluating the tumor-specific cell-free DNA (cfDNA) methylation status of preoperative and postoperative blood samples. We then comprehensively compared the tumor-informed MRD model with tumor-naïve global methylation status (MethylMean) and tumor-informed ctDNA mutation status. Lastly, we illustrated the feasibility of methylation-based cancer surveillance with timMRD in the MEDAL cohort and validated it in an independent cohort from the DYNAMIC study with survival outcome [2].

## Methods

### Patient selection

Patients were prospectively enrolled between August 2018 and July 2019. The protocol of this study was previously published [15]. The inclusion/exclusion criteria were described in the Additional file 1: Supplementary Methods. The recruited patients were followed up every three to six months by experienced thoracic surgeons. An independent cohort from a previously published study (DYNAMIC) [2] comprising 36 patients with adequate/remaining blood and tissue samples were included for validation.

### Study design

**Fig. 1:**
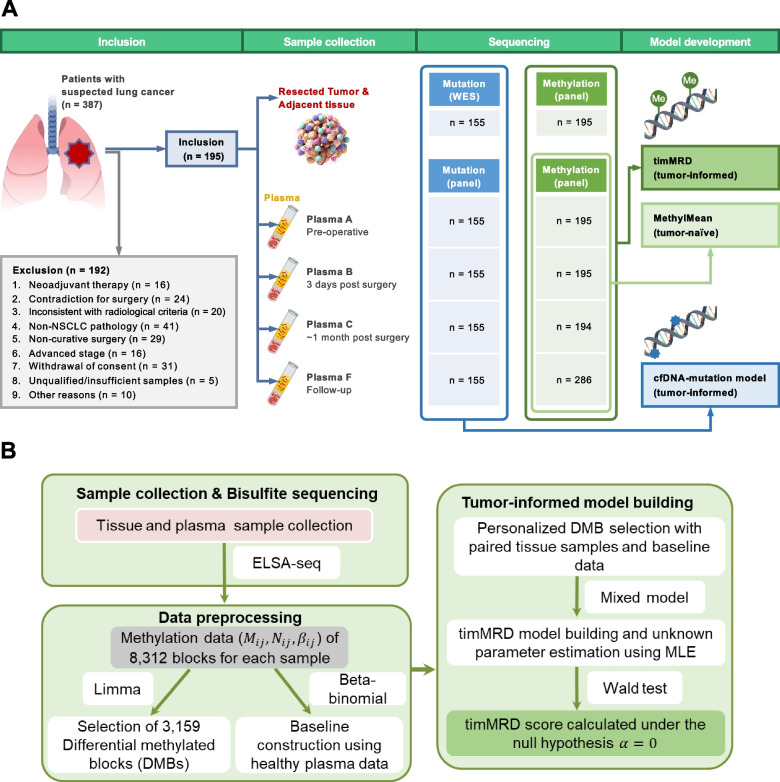
Workflow of the study design and methylation-based analysis. Flow diagram illustrating the study design (See Methods section). Plasma C was collected at a median of 36 days due to discrepancy in patient follow-up visits; however, all plasma samples were collected before adjuvant therapy was administered

The study design is illustrated in Fig. 1. Tissue samples were collected during surgery, and normal tissues were obtained between 2 to 5 cm from the surgical margin, with the most distant sample used for further analysis. Blood samples were collected before surgery (Plasma A), 3-days post-surgery (Plasma B), approximately 1-month post-surgery (Plasma C) before adjuvant therapy, and during subsequent follow-up (Plasma F). A total of 870 longitudinal plasma samples were assayed for somatic mutation and methylation status. The abundance of ctDNA mutations was reflected as the maximum allele frequency (maxAF) and defined as the highest fraction of the mutant allele detected in each sample. The average cfDNA methylation level was calculated as MethylMean. Meanwhile, timMRD-score explicitly illustrated in Additional file 2: Figure S1 was calculated for each plasma sample by a statistical model of the methylation status of paired tumor and normal tissues (See Methods timMRD model).

### Next-generation sequencing

DNA isolation, library preparation procedures for the unique molecular identifier (UMI)-based targeted sequencing and bisulfite targeted sequencing, and corresponding data analyses were performed according to previously published protocols [15–18]. The performance of our UMI-based targeted sequencing was analytically validated in a report by the FDA-led sequencing quality control phase 2 (SEQC2) oncopanel sequencing working group [18] and demonstrated the lowest false positive rate at 25 ng input among 5 UMI-based hybrid-capture ctDNA assays. The library preparation for bisulfite targeted sequencing was performed following our optimized protocol for cfDNA methylation-based assay (ELSA-seq) [17]. ELSA-seq plus a machine learning classifier enabled the detection of tumor-derived signals at dilution factor as low as 1:10,000. Differentially methylated blocks (DMBs) were stratified as described in the Additional file 1: Supplementary Methods.

### Unique molecular identifier (UMI)-based targeted ultra-deep mutation sequencing

As described previously [17], acoustically sheared tissue DNA and cfDNA fragments between 200 and 400 bp were purified, end-repaired, and A-tailed. cfDNA were ligated with UMI-containing sequencing adapters designed by Burning Rock Biotech. Tissue DNA were processed accordingly with non-UMI sequencing adapters. After purification using Agencourt AMPure XP Kit (Beckman Coulter, CA, USA), the adapter-ligated DNA were hybridized with capture probes baits, hybrid-selected with magnetic beads, and amplified. Target capture was performed using a commercial panel with RNA baits designed for 168 lung cancer-related genes, spanning 273 kb of the human genome (Lung Plasma UMI; Burning Rock Biotech, Guangzhou, China), which was validated in a study by the FDA-led sequencing quality control phase 2 (SEQC2) oncopanel sequencing working group [18]. The indexed samples were sequenced on Illumina NovaSeq 6000 (Illumina, San Diego, CA, USA) with 2 × 150 bp and target sequencing depth of 1,000 × for tissue samples and 30,000 × for plasma samples. Sequencing data were analyzed using proprietary computational algorithms optimized for somatic variant calling.

### TimMRD model assumption and parameter estimation

Let *N*_*ij*_ be the total number of CpG sites detected in *j*-th differentially methylated blocks (DMB) of *i*-th blood sample, *M*_*ij*_ be the total number of methylated CpG sites detected in *j*-th DMB of *i*-th blood sample. We assume that cfDNA methylation sequencing data (${M}_{ij},{N}_{ij}$) follows a mixed beta-binomial distribution defined in equation (Eq. 1)

1 $${\mathrm{M}}_{\mathrm{ij}}|{\mathrm{N}}_{\mathrm{ij}},{\upbeta }_{\mathrm{ij}} \sim \mathrm{ Binomial}({\mathrm{N}}_{\mathrm{ij}},{\upbeta }_{\mathrm{ij}})$$

where parameter β_ij_ denotes the probability of methylation occurrence. Since the cfDNA isolated from the patient's blood contains DNA from both lung tumor cells (ctDNA) and non-tumor/normal cells, we can deconvolute β_ij_ as:

2 $$\beta_{ij}=\alpha_i\beta_{ij}{}^{(T)}+\gamma_i\beta_{ij}{}^{(N)}+\left(1-\alpha_i-\gamma_i\right)\;\beta_{ij}{}^{(0)}$$

where ${{\upbeta }_{\mathrm{ij}}}^{(\mathrm{T})}$ and ${{\upbeta }_{\mathrm{ij}}}^{(\mathrm{N})}$ represent corresponding methylation levels of the *j*-th DMB from the patient's tumor and matched tumor-adjacent normal tissue sample, parameters ${\mathrm{\alpha }}_{\mathrm{i}}$ and $0\le {\alpha }_{i},{ \gamma }_{i}<1$ represent proportions of ctDNA and cfDNA in the blood, respectively, and satisfy the condition ${\mathrm{\alpha }}_{\mathrm{i}}+{\upgamma }_{\mathrm{i}}<1$.$\;\beta_{ij}{}^{(0)}$ is the normal methylation level of DNA from other cells in the blood. Since it is impossible to collect blood samples of cancer patients in their healthy state, we used sequencing data derived from blood samples of a healthy cohort (described above) to fit the prior distribution of $\beta_{ij}{}^{(0)}$*.* Based on the previous studies [19, 20], $\beta_{ij}{}^{(0)}$  was assumed to follow a beta distribution with shape parameters ${(p}_{j}, {q}_{j})$. According to the distribution assumption (Eq. 1), the mixed beta-binomial distribution for the patient plasma methylation data can then be derived given the unknown parameters {${\alpha }_{i},{ \gamma }_{i}$} with the log-likelihood functions summarized as:

3 $$l\left({\alpha }_{i},{\gamma }_{i}\right)={\sum }_{j=1}^{\mathrm{m}}\mathrm{log}({l}_{ij}\left({\alpha }_{i},{\gamma }_{i};{M}_{ij},{N}_{ij}\right))$$

where

4 $$l_{ij}\left(\alpha_i,\gamma_i;M_{ij},N_{ij}\right)=\int_0^1\;f\left(M_{ij}\vert N_{ij},\beta_{ij}\right)g\left(\beta_{ij}\right)d\beta_{ij}$$

wherein *f* and *g* denote the density functions of the binomial and beta distributions accordingly. Using$\beta_{ij}^{(0)}$ as a prior together with estimated distribution parameter values *p*_*j*_ and *q*_*j*_, and expressing it from the Eq. 2, we can rewrite the latter equation as:

5 $${l}_{ij}({\alpha }_{i},{\gamma }_{i};{M}_{ij},{N}_{ij}) ={\int }_{0}^{1}\left(\genfrac{}{}{0pt}{}{{M}_{ij}}{{N}_{ij}}\right){{{\beta }_{ij}}^{{M}_{ij}}\left(1-{\beta }_{ij}\right)}^{{N}_{ij}-{M}_{ij}}g(\frac{{\beta }_{ij}-{\alpha }_{i}{{\beta }_{ij}}^{\left(T\right)}-{\gamma }_{i}{{\beta }_{ij}}^{(N)}}{1-{\alpha }_{i}-{\gamma }_{i}},{p}_{j}, {q}_{j})d{\beta }_{ij}$$

In Eq. 3, Eq 4, Eq. 5, the unknown parameters ${\alpha }_{i},{\gamma }_{i}$ satisfy the conditions ${\alpha }_{i}\ge 0, {\gamma }_{i}\ge 0$ and ${\alpha }_{i}+{\gamma }_{i}<1$.

To improve the identifiability of parameters in the function, patient-specific DMBs were first selected using personal data for tissues, and prior information of $\beta_{ij}{}^{(0)}$ . ${\alpha }_{i}$ and ${\gamma }_{i}$ were then estimated by applying the maximum likelihood estimation (MLE) method, with ${\alpha }_{i}$ indicating the malignancy density (MD) ratio. The Wald statistic under the null hypothesis: ${\alpha }_{i}=0$ was defined as timMRD-score which could be used to assess the existence of ctDNA. Following chi-square distribution with 1 degree of freedom, 5.412 (98^th^ quantile) was selected as the threshold for model prediction. Samples with timMRD-score > 5.412 were defined as timMRD-high, whereas samples with timMRD-score ≤ 5.412 were defined as timMRD-low.

### Statistics

Data were analyzed using the R software package (R version 3.4.0). A range of appropriate statistical hypothesis testing technique were applied, including Student’s t-test, Fisher’s exact test, Kruskal–Wallis H test, two-tailed Wilcoxon signed-rank test, Spearman rank-order, or Pearson correlation test, to identify potential significant differences among the groups. Disease-free survival (DFS) was defined as the days from surgery to radiologically confirmed recurrence, or death from any cause when no evidence of relapse was recorded. Survival analyses were performed using the Kaplan–Meier method with log-rank test. Hazard ratios (HR) with corresponding 95% confidence intervals (CI) were calculated using univariable Cox proportional-hazards regression model. Multivariable analyses were also performed using Cox proportional-hazards regression model. *P* values < 0.05 were considered statistically significant.

### Study approval

This study was performed according to the Declaration of Helsinki in 1964 and its current amendments. The study protocol was approved by the Medical Ethics Committee of the Peking University People’s Hospital (approval number: 2018PHB077–01). Written informed consent was obtained from every patient before study enrollment.

## Results

### Patient characteristics and tumor-informed methylation analysis

Of the 387 consecutive patients with suspected NSCLC, a total of 195 patients (stage I-II 165, 84.6%) were included in the MEDAL cohort. The clinicopathological features of the MEDAL cohort were comparable to that of the independent DYNAMIC cohort [2] (Table 1; Additional file 3: Table S1). Figure 1 provides an overview of the study design.

**Table 1:** Baseline clinicopathologic features of the two independent cohorts

Clinicopathologic features	MEDAL cohort *n* = 195 n (%)	DYNAMIC cohort *n* = 36 n (%)	*P*-value
**Age, years (median, range)**	63 (35–84)	62.5 (39–78)	0.67
**Sex**			0.93
**Female**	82 (42.1%)	16 (44.4%)	
**Male**	113 (57.9%)	20 (55.6%)	
**Smoking history**			0.76
** Non-smoker**	116 (59.5%)	23 (63.9%)	
** Smoker**	79 (40.5%)	13 (36.1%)	
**Histology**			0.50
** Lung adenocarcinoma**	130 (66.7%)	28 (77.8%)	
** Lung squamous cell carcinoma**	51 (26.1%)	7 (19.4%)	
** Other NSCLC**	14 (7.2%)	1 (2.8%)	
**Pathological stage**			0.28
** I**	128 (65.6%)	28 (77.8%)	
** II**	37 (19.0%)	3 (8.3%)	
** ≥ IIIA**	30 (15.4%)	5 (13.9%)	
**Tumor diameter, cm (median, range)**	2.5 (1.0–8.2)	2.3 (1.1–9.3)	0.77
**Visceral pleural involvement**			0.63
** With**	45 (23.1%)	10 (27.8%)	
**Without**	144 (73.8%)	24 (66.7%)	
** No data**	6 (3.1%)	2 (5.5%)	
**Duration of surgical procedure, minutes (median, range)**	140 (60–325)	132.5 (60–300)	0.52
**Surgical method**			1.00
** Lobectomy**	175 (89.7%)	33 (91.7%)	
** Wedge resection**	20 (10.3%)	3 (8.3%)	
**Devascularization technique performed during surgery**			0.50
** Arterial first**	134 (68.7%)	27 (75.0%)	
** Venous first**	49 (25.1%)	6 (16.7%)	
** No data**	12 (6.2%)	3 (8.3%)	
**Adjuvant therapy**			0.54
** With**	55 (28.2%)	8 (22.2%)	
**Chemotherapy**	47 (24.1%)	7 (19.4%)	
** Targeted therapy**	5 (2.6%)	1 (2.8%)	
** Immunotherapy**	1 (0.5%)	0 (0.0%)	
** Radiotherapy**	2 (1.0%)	0 (0.0%)	
** Without**	140 (71.8%)	28 (77.8%)	

*Abbreviations*: *NSCLC* non-small-cell lung cancer

**Fig. 2:**
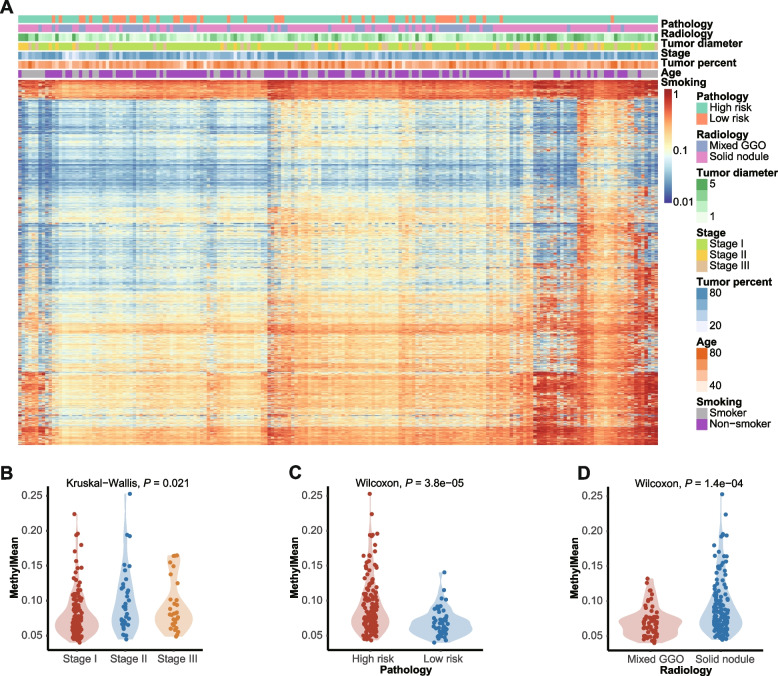
Baseline global methylation status of tumor samples are associated with clinicopathologic features. **A** Unsupervised hierarchical clustering of the hypermethylation ratios of tumor tissues. The clinical details of each patient were annotated above including pathologically-evaluated risk of recurrence, radiological features, tumor diameter, pathological stage, tumor cell percentage of the tumor sample, age, and smoking status. **B-D** Violin plots comparing the distribution of the hypermethylation ratio according to the pathological stage (**B**), pathological risk (**C**), and radiological features characterized either as solid nodule (solid) or mixed ground-glass opacity (mix) (**D**). Kruskal–Wallis H test (B) or Wilcoxon signed-rank test (C-D) was performed to compare the difference between groups. Statistical significance was defined as *P*-value < 0.05

Although the cfDNA input for targeted bisulfite sequencing was variable, particularly for Plasma B (range, 6–31 ng; Additional file 2: Figure S2A), the methylation levels for all CpG blocks were evenly distributed with minor variation across time-points for the blood samples, indicating the quality and consistency of bisulfite sequencing (Additional file 2: Figure S2B). The methylation levels of the tumor samples had very distinct distributions in comparison with the paired tumor-adjacent normal samples obtained 2 or 5 cm away from the tumor margin (Additional file 2: Figure S2C). Clustering analyses revealed the distinct methylation profiles in tumor tissues, tumor-adjacent normal tissues, and blood samples; however, blood samples across time-points were indistinguishable (Fig. 2A; Additional file 2: Figure S2D). The heterogeneous methylation profiles of the tumors and blood samples suggested the potential benefits of a tissue-informed approach in blood-based methylation analysis (Additional file 2: Figure S2D). The bisulfite conversion metrics for non-CpG sites revealed consistent and satisfactory bisulfite conversion rates for blood samples (Additional file 2: Figure S2E) and tissue samples (Additional file 2: Figure S2F). The timMRD-scores of blood samples collected at different time-points were not correlated with the tumor cell percentage of tumor tissue samples (Additional file 2: Figure S2G), indicating the robustness of timMRD-scores.

We further explored the global methylation profiles of tumor tissues. The global methylation profile of tumor tissues was distinct from tumor-adjacent normal tissues and plasma samples (Additional file 2: Figure S3). The direct association between global methylation profiles and disease characteristics was demonstrated by unsupervised hierarchical clustering of tumor tissue samples (Fig. 2A) and subgroup analyses (Figs. 2B-2D). Higher levels of MethylMean were associated with higher pathological stages (*P* = 0.021; Fig. 2B), and high-risk histological subtypes (*P* < 0.001, Fig. 2C) and radiological features (*P* < 0.001; Fig. 2D, see Additional file 1: Supplementary Methods for definitions). Additional file 4: Data S1- Additional file 7: Data S4 summarizes the detailed clinical, mutational, and methylation information of each sample.

### Characterization of baseline ctDNA mutation and methylation status

Of the 155 patients evaluable for tumor-informed ctDNA mutation status at baseline, 47 (30.3%) had detectable ctDNA mutations in the baseline plasma samples (Additional file 2: Figure S4). *TP53* (61.7%, 29/47) was the most frequently mutated gene, followed by *EGFR* (14.9%, 7/47). Baseline cfDNA methylation status was indicated as either the tumor-naïve MethylMean or the tumor-informed timMRD-score (Methods). Listed by descending order of timMRD-scores, we observed an obvious cluster of mutation-positive cases with high timMRD-scores (Additional file 2: Figure S4).

To validate the accuracy and robustness of the timMRD model, a series of in vitro dilution experiments and single-parameter and paired-parameter numerical simulations were performed as described in the Additional file 1: Supplementary Methods. First, we evaluated the accuracy of timMRD model using serial dilution experiments with lung cancer cell line. These dilution experiments demonstrated a 95% accurate detection of methylation signals at tumor dilution factor as low as 1:5,000 or a tumor fraction of 0.0002, which had timMRD-scores at the cutoff. This finding indicates the accuracy and limit of detection for the timMRD model (Additional file 2: Figure S5A). Next, we performed numerical simulations to evaluate whether timMRD-score can identify the quantity of tumor-derived DNA represented by the simulated cfDNA tumor fraction. As shown in Additional file 2: Figure S5B, timMRD-scores were directly proportional to the simulated cfDNA tumor fractions, indicating high accuracy even at a tumor fraction as low as 0.01%. The paired-parameter simulations demonstrated that timMRD-scores were only slightly affected by the proportion of normal lung tissues when the simulated tumor fraction is > 0.1%, indicating a marginal to negligible contribution of DNA from non-tumor cells to the timMRD-score estimation (Additional file 2: Figure S5C).

**Fig. 3:**
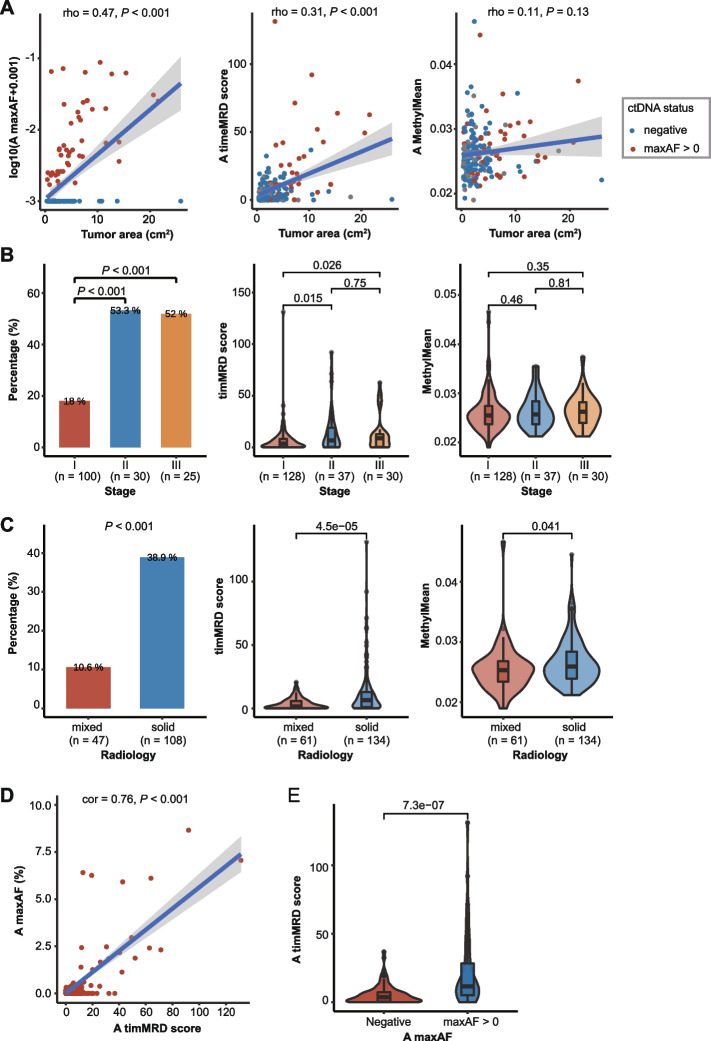
Tumor-informed cfDNA methylation scores at baseline are positively associated with disease burden and the presence of ctDNA. **A** Relationship between tumor area (expressed as cm^2^) and maxAF of mutations (left panel), timMRD score (middle panel), and MethylMean (right panel). The gray shadow denotes 95% confidence intervals. The colors of the dots indicate ctDNA status at baseline, wherein blue represents undetectable ctDNA mutations (*n* = 108), and red represents positive ctDNA status as samples with maxAF > 0 (*n* = 47). Spearman rank-order correlation test was performed to analyze the correlation between the molecular marker and tumor area. **B-C** Distribution of somatic mutations reflected as mutation positive rates (left), timMRD scores (middle), and MethylMean (right) according to pathological stages (**B**); and radiological features (**C**). Pairwise comparisons were performed using Fisher’s exact test or Wilcoxon signed-rank test. Statistical significance was defined as *P*-value < 0.05. **D** Relationship between timMRD score and tumor-informed somatic mutation status (maxAF) for 47 patients who were ctDNA-positive at baseline. The blue line denotes the best-fitting line. The gray shadow denotes 95% confidence intervals. Pearson correlation test was performed to analyze the correlation between the timMRD-score and maxAF of ctDNA mutations. **E** Comparison of the timMRD scores between patients with undetectable and detectable ctDNA mutations. Wilcoxon signed-rank test was performed to compare the difference between groups. Statistical significance was defined as *P*-value < 0.05

Our data revealed no association between MethylMean and tumor area (Fig. 3A) or diameter (Additional file 2: Figure S6A). In contrast, the timMRD-score (*P* < 0.001, Fig. 3A; *P* < 0.001, Additional file 2: Figure S6A) and the abundance of ctDNA mutations (*P* < 0.001, Fig. 3A; *P* < 0.001, Additional file 2: Figure S6A) were positively associated with larger tumor areas and diameters. Baseline timMRD-scores were significantly higher in patients in more advanced stage (Fig. 3B), solid nodules (*P* < 0.001, Fig. 3C), and smokers (*P* < 0.001, Additional file 2: Figure S6B). The sensitivity of baseline timMRD model in the MEDAL and DYNAMIC cohorts were 40.6% (52/128) and 39.3% (11/28) for stage I, respectively. Consistently, tumor-informed ctDNA mutation status showed the same trend as the timMRD-scores in patients with advanced stage, solid nodules, and smokers (*P* < 0.001, Fig. 3B; *P* < 0.001, Fig. 3C; *P* < 0.001, Additional file 2: Figure S6B) but was poorly detected in adenocarcinoma (*P* < 0.001, Additional file 2: Figure S6C). In contrast, no statistical difference was observed for baseline MethylMean values, except for smoking status (Spearman’s correlation; Fig. 3A-C; Additional file 2: Figure S6B-C). Moreover, the baseline timMRD-scores were highly correlated with the maxAF in ctDNA-positive samples (Pearson correlation *r* = 0.76, *P* < 0.001; Fig. 3D). The timMRD-score could distinguish ctDNA-positive (maxAF > 0) from ctDNA-negative samples (*P* < 0.001, Fig. 3E). A majority of patients who were baseline ctDNA mutation-positive also had high timMRD-scores (74.5%, 35/47).

These results demonstrate that the baseline timMRD-scores could reflect the tumor burden and invasiveness. TimMRD-score was also positively correlated with the abundance of tumor-specific mutations in cfDNA, indicating its potential in identifying MRD at the molecular level.

### Dynamics of cfDNA methylation in the perioperative period associated with presence/absence of residual disease

We further explored the dynamics of cfDNA methylation during the perioperative period (Plasma A to C, Additional file 2: Figure S7A). No dramatic change was observed in MethylMean at perioperative time-points (*P* = 0.037, Additional file 2: Figure S7A). Meanwhile, the abundance of ctDNA mutations decreased rapidly after curative resection, with most baseline mutation-positive cases having zero maxAF for Plasma C (*P* < 0.001; Additional file 2: Figure S7B). Also, the timMRD-score revealed a declining pattern for Plasma A to C (*P* = 0.007, Additional file 2: Figure S7C). The observed differences in the timMRD-score were not affected by the surgical procedures (Additional file 2: Figure S8). Consistent with the baseline samples, the timMRD-scores were positively correlated with the maxAF of ctDNA mutations at all time-points (Pearson correlation *r* = 0.46, *P* < 0.001; Additional file 2: Figure S9).

Compared to patients with no evidence of disease, the relapsed patients revealed a significantly higher mutation positive rate for Plasma B (*P* < 0.001) and Plasma C (*P* < 0.001, Additional file 2: Figure S10A). Similarly, timMRD-scores were significantly higher in samples of relapsed patients for Plasma B (*P* = 0.049) and Plasma C (*P* = 0.023, Additional file 2: Figure S10B). In contrast, MethylMean did not reflect these dynamic changes (*P* > 0.1, Additional file 2: Figure S10C).

Consistent with somatic mutation status, the timMRD-score revealed a rational dynamic pattern during perioperative periods and was significantly elevated in relapsed patients at postoperative time-points, indicating its potential utility in MRD detection.

### TimMRD can be used for cfDNA methylation-based prognostic prediction

**Fig. 4:**
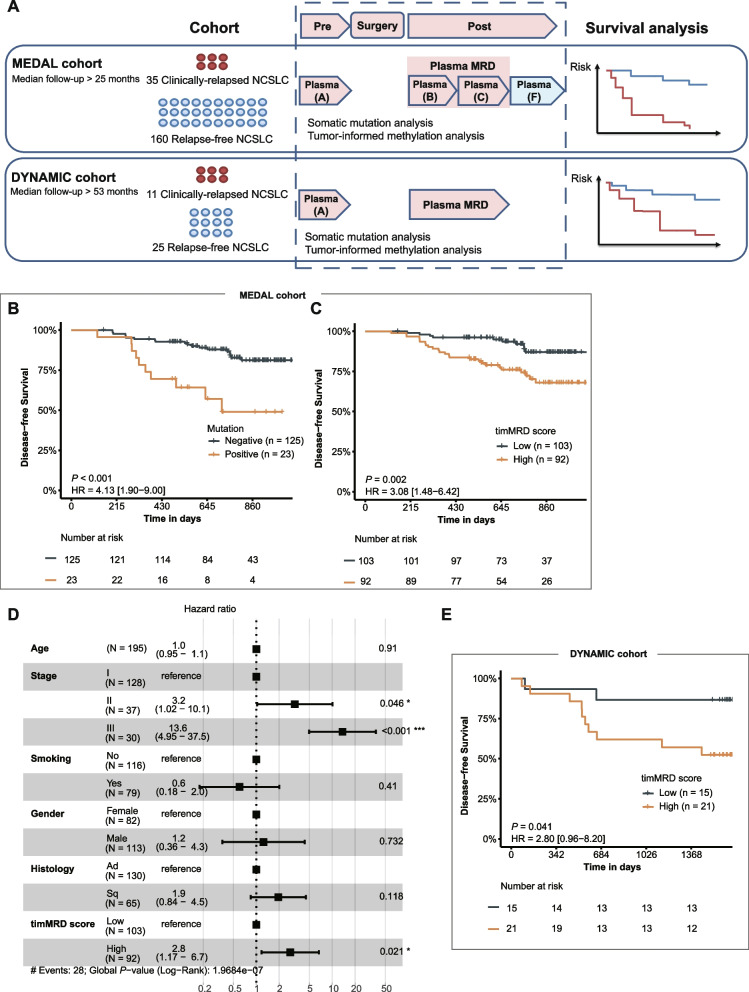
Risk assessment by somatic mutation and timMRD score in two independent cohorts. **A** Schematic diagram illustrating the analytical procedures in the MEDAL cohort (n = 195) and the DYNAMIC cohort (n = 36). **B-C, E** Kaplan–Meier analysis of disease-free survival (DFS, expressed in days) according to ctDNA mutation status (**B**) and timMRD scores (**C**) at postoperative time points (Plasma B/C) of MEDAL (**B, C**) and DYNAMIC (**E**) cohorts. The patients were classified according to their tumor-informed ctDNA mutation status and timMRD scores of Plasma B or Plasma C. The DFS of each patient was computed from the date of surgery until radiological confirmation of disease relapse. Tick marks indicate patients who were disease-free at data cut-off date. The risk table below the KM plot summarizes the number of patients included per time point. KM survival analysis was performed with log-rank statistics to compare the survival between the two subgroups. Hazard ratio (HR) and corresponding 95% confidence intervals (CI) were computed using univariable Cox proportional-hazards regression model. **D** Forest plot summarizing the results of the multivariable analysis for DFS of the MEDAL cohort. Multivariable Cox proportional-hazards regression model was performed to compute the HR and corresponding 95% CI

To explore the capability of timMRD for prognostic prediction, we analyzed the survival data of the MEDAL cohort and validated it in the independent DYNAMIC cohort [2] (Fig. 4A). In the MEDAL cohort, 35 patients exhibited tumor recurrence, with a median follow-up of two years (25 months) (Fig. 4A). The DFS was significantly shorter for mutation-positive patients for Plasma B/C (HR: 4.13, 95% CI: 1.90–9.00, *P* < 0.001; Fig. 4B; see Additional file 2: Figure S11A for Plasma B and C separately), albeit considerably lower ctDNA mutation rates (n = 23). Based on the 98^th^ percentile of the chi-square distribution for timMRD-scores, we used a cutoff of 5.412 to stratify the patients into two groups (low vs. high timMRD-score; see Additional file 1: Supplementary Methods for details). DFS was also significantly shorter among the patients with higher timMRD-score for Plasma B/C (HR: 3.08, 95% CI: 1.48–6.42, *P* = 0.002; Fig. 4C; see Additional file 2: Figure S11B for Plasma B and C separately) and two-years post-surgery (HR: 2.50, 95% CI: 1.36–4.57, *P* = 0.002; Additional file 2: Figure S12A). Moreover, multivariable Cox regression analysis also identified high postoperative timMRD-score as an independent predictive factor for worse DFS (HR: 2.80, 95% CI: 1.17–6.70, *P* = 0.021; Fig. 4D).

The DYNAMIC cohort consists of 36 patients with adequate archival blood samples, with a median follow-up of > 4.4 years and comparable clinicopathological characteristics with the MEDAL cohort (Additional file 3: Table S1). Using the same cutoff, patients with high postoperative timMRD-scores consistently had significantly shorter DFS (HR: 2.80, 95% CI: 0.96–8.20, *P* = 0.041; Fig. 4E).

We also explored if these observations could be identified in patients with lower disease burden in the MEDAL cohort. For Plasma B/C, ctDNA mutations were detected in only 13.0% (13/100) of the evaluable patients with stage I tumors and 16.7% (17/102) of patients with adenocarcinoma histology, whereas two of the relapsed patients with stage I (28.6%, 2/7) and only 31.2% (5/16) of relapsed patients with adenocarcinoma were ctDNA mutation-positive. Contrastingly, more relapsed patients with stage I (85.7%, 6/7) and adenocarcinoma (68.8%, 11/16) had high timMRD-scores for Plasma B/C. Prognostication at two years post-surgery using timMRD-scores for Plasma B/C also revealed the association between shorter DFS and higher timMRD-scores in patients with stage I (*P* = 0.047, Additional file 2: Figure S12B) and adenocarcinoma (*P* = 0.029, Additional file 2: Figure S12C). Moreover, among the 14 relapsed patients who were ctDNA-negative at baseline, only a patient was detected with ctDNA mutations from Plasma B/C, while timMRD-scores were high for 64.3% (9/14) of relapsed patients. Two-year prognostication demonstrated a trend of shorter DFS for baseline ctDNA-negative patients with high postoperative timMRD-score (HR: 2.77, 95% CI: 0.91–8.43, *P* = 0.050; Additional file 2: Figure S12D).

These results from two independent cohorts indicates the feasibility of prognostication by timMRD-scores at early postoperative time-points and its advantage in patients with low tumor burden.

### TimMRD-scores are applicable for methylation-based surveillance strategy in lung cancer

**Fig. 5:**
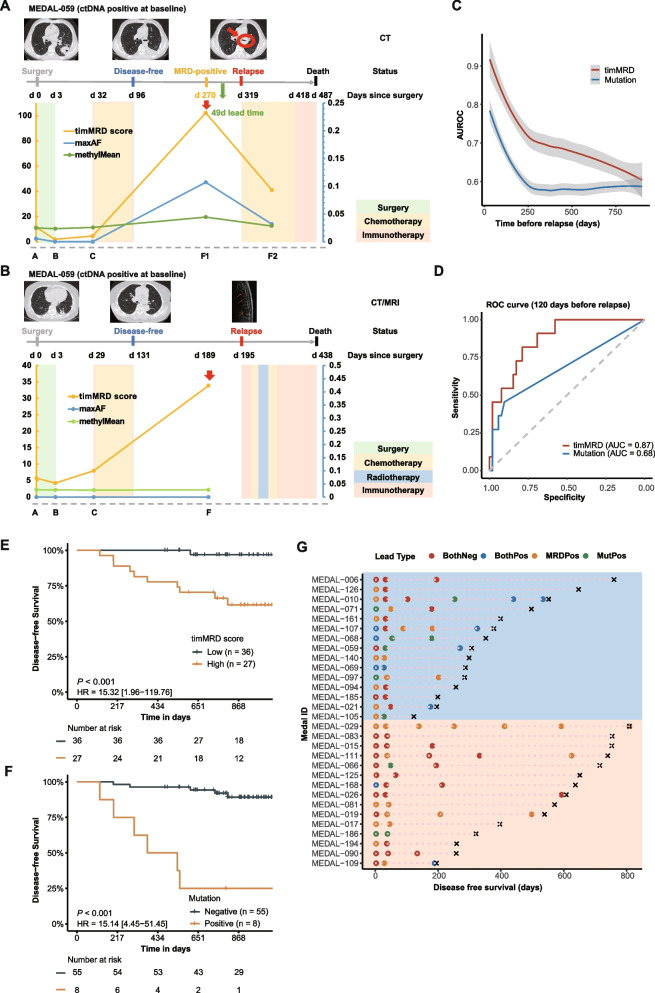
The performance of somatic mutation and timMRD score in prognostication. **A-B** Graphical summary of the medical history and the dynamic monitoring of ctDNA mutation and cfDNA methylation profile for Patients MEDAL-059 (**A**) and MEDAL-109 (**B**). Top images show the radiological imaging at different time points and the treatment timeline for each patient. Bottom plot summarizes the dynamics of the ctDNA mutations, MethylMean, and timMRD scores at different perioperative and postoperative time points. The colored shading corresponds to the treatment modalities received by the patient at the specified time point. **C-D** Diagnostic performance of timMRD score and tumor-informed ctDNA mutation positive rate in identifying relapsed patients. **C**. Area under the receiver operating characteristic (AUROC) curve illustrating the performance of timMRD score and tumor-informed ctDNA mutation positive rate over time before radiological confirmation of disease relapse. **D** Receiver operating characteristic (ROC) curves plotting the specificity and sensitivity for timMRD score and tumor-informed ctDNA mutation positive rate revealing the area under the curve (AUC) at 120 days before relapse. **E–F** Kaplan Meier analysis of disease-free survival (DFS, expressed in days) of timMRD scores (**E**) and ctDNA mutation status (**F**) evaluated using the last postoperative follow-up sample 120 days before relapse. The patients were classified according to their timMRD scores for the last follow-up sample. The DFS of each patient was computed from the date of surgery until radiological confirmation of disease relapse. Tick marks indicate patients who were disease-free at data cut-off date. The risk table below the KM plot summarizes the number of patients included per time point. KM survival analysis was performed with log-rank statistics to compare the survival between the two subgroups. Hazard ratio (HR) and corresponding 95% confidence intervals (CI) were computed using univariable Cox proportional-hazards regression model. **G** Dynamic postoperative analysis of timMRD scores and mutation detection in 30 relapsed patients evaluable for tumor-informed somatic mutation status reveals the lead time before radiological confirmation of relapse for each molecular assay. The data was arranged in descending order of DFS and grouped according to ctDNA status at baseline, wherein the top 15 patients were ctDNA-positive at baseline and the bottom 15 patients were ctDNA-negative at baseline. The first data points correspond to results from Plasma B. Colored dots represent the molecular assay that detected the molecular residual disease. BothPos denotes positive status for both timMRD-score (high) and ctDNA mutation; MRDPos denotes timMRD-score high status; MutPos denotes positive tumor-informed ctDNA mutation status. Time of disease relapse is marked with ×

Next, we hypothesized that the dynamic changes in timMRD-scores may provide an innovative strategy for cancer surveillance. As shown in Fig. 5A, the timMRD-score and maxAF of somatic mutations decreased rapidly for Plasma B and C after patient MEDAL-059, ctDNA-positive at baseline, received curative surgery. Thereafter, the significant elevations of timMRD-score and maxAF were observed concomitantly at 270 days post-surgery, with a lead time of 49 days before clinical confirmation of recurrence. Figure 5B illustrated patient MEDAL-109, with no ctDNA mutation detected at any perioperative time-point, who had a remarkable elevation of timMRD-score for Plasma B (4.244), Plasma C (7.978), and Plasma F (33.865) at 189 days after surgery. Dynamic analysis of another two relapsed patients consistently demonstrated elevated timMRD-scores with or without ctDNA mutations before radiological detection of relapse (Additional file 2: Figure S13). No specific pattern was observed in the MethylMean of the relapsed patients (Fig. 5A-B, Additional file 2: Figure S13).

We next compared the predictive accuracy of tumor-informed ctDNA mutations and timMRD-scores. At various postoperative time-points before relapse, timMRD-scores demonstrated significantly better performance in predicting relapse than ctDNA mutation in almost all the time-points (Fig. 5C). Previous three studies focusing on lung cancer MRD detection have demonstrated the accuracy of both tumor-naïve and tumor-informed ctDNA-based MRD detection in predicting recurrence with an average lead time of 127 ± 42 days before clinical diagnosis (Additional file 3: Table S2). Hence, we compared the performance of timMRD-score and tumor-informed ctDNA mutation status at 120 days before clinical diagnosis of disease recurrence in the MEDAL cohort. Although ctDNA mutations had higher specificity than the timMRD-scores (90.4% vs. 67.3%); the assay sensitivity for timMRD-scores increased over 70% compared with ctDNA mutations (90.9% vs. 45.5%). Note that some patients may have clinically undetected radiological recurrence, which may underestimate the specificity for both. The area under the curves (AUC) for the timMRD-scores was 0.87, and 0.68 for ctDNA mutations (Fig. 5D). At the same sensitivity level of 45.5% for both assays, timMRD model using a cutoff at 99.99^th^ percentile only had 1 false positive call, yielding a higher specificity (98.1% vs. 90.4%) than tumor-informed mutation status. At the same specificity level of 90.4% for both assays, the sensitivity (54.5% vs. 45.5%) and negative predictive value (90.4% vs. 88.7%) were consistently higher for timMRD-score using a cutoff at 99.95^th^ percentile as compared with tumor-informed mutation status (Additional file 3: Table S3). DFS was significantly shorter for patients with high timMRD-score evaluated using the last follow-up sample approximately 120 days before relapse (HR:15.32, 95% CI: 1.96–119.76; *P* < 0.001; Fig. 5E). Only one relapsed patient had low timMRD-score, resulting in a negative predictive value of 97.2%. DFS was also significantly shorter for patients with positive ctDNA mutation status evaluated using the last follow-up sample approximately 120 days before relapse (HR:15.14, 95% CI: 4.45–51.45; *P* < 0.001; Fig. 5F). Figure 5G summarizes the mutation and methylation status during follow-up of 30 relapsed patients stratified according to baseline tumor-informed ctDNA mutation status as positive (blue) and negative (orange) groups. The average lead time before confirmation of disease recurrence was 303 days for timMRD-score and 137 days for tumor-informed ctDNA mutation status compared with radiological diagnosis. As expected, baseline ctDNA mutation status was positively associated with significantly shorter DFS (median DFS 10.3 vs. 20.5 months; *P* = 0.031 Wilcoxon) and the detection of ctDNA mutation at any follow-up time-point before confirmation of relapse (baseline positive vs. negative, 56.3% [9/16] vs. 7.1% [1/14], *P* = 0.007). At the last follow-up time-point before relapse, ctDNA mutation positivity was significantly higher among relapsed patients with positive than negative baseline ctDNA mutation status (68.8% [11/16] vs. 14.3% [2/14], *P* = 0.004). In contrast, both ctDNA-positive and ctDNA-negative groups had similarly high timMRD-scores for Plasma B/C (68.8% [11/16] vs. 64.3% [9/14], *P* = 0.350) or at the last follow-up before relapse (75.0% [12/16] vs. 71.4% [10/14], *P* = 0.694). ctDNA mutation status and timMRD-score yielded similar sensitivity among relapsed patients with baseline ctDNA-positive status (68.8% vs. 75.0%); whereas timMRD-score was more accurate than ctDNA mutation status among relapsed patients with baseline ctDNA-negative status (64.3% vs. 14.3%). These results suggest the reliability of timMRD-based postoperative monitoring, particularly in patients with negative baseline ctDNA status.

## Discussion

The abundance of circulating cfDNA originating from non-cancerous cells could significantly obscure the small genetic fragments released by solid tumors, resulting in the diluted ctDNA concentration, particularly in the smaller tumor bulk of early-stage cancers. This low ctDNA concentration makes it challenging to detect postoperative MRD using ctDNA mutations [21]. To the best of our knowledge, this is the first prospective study to investigate the feasibility of methylation-based MRD detection in early-stage malignancies (NCT03634826) [15] and adopt a personalized approach to MRD analysis using a methylation assay.

Tumor-informed models are recognized to improve sensitivity and accuracy in detecting low-frequency somatic mutations from blood samples [5, 22, 23]. Since radical resection enables the collection of tissue samples, both genetic and epigenetic analysis can benefit from the personalized tumor-informed approach, providing a unique advantage to the field of postoperative MRD monitoring. Our study comprehensively elucidated the DMBs through targeted bisulfite sequencing of 80,672 CpG sites. As described in the Additional file 1: Supplementary Methods section and the recent publication by Liang et al. [17], these 80,672 CpG sites were identified as lung cancer-specific based on publicly available 450 k microarray-derived methylome data from The Cancer Genome Atlas [24]. These CpG sites were first clustered into 8,312 methylation blocks and further compared with tumor-adjacent normal tissue samples from the 195 patients in the MEDAL cohort, which identified the 3,159 DMBs in lung tumors. Patient-specific DMBs were then selected from the 3,159 tumor-specific DMBs, which guaranteed the robustness of the individualized analysis of blood samples at different time-points. The cfDNA methylome data were analyzed using this innovative statistical model that integrates the methylation data gathered from paired tumor and normal tissue samples. The use of a tumor-informed approach in analyzing the methylation data from a fixed panel could minimize the contribution of epigenetic heterogeneity and is considerably cost-effective and applicable for clinical practice.

We have observed the positive association between timMRD-score and the abundance of ctDNA mutations and disease characteristics as indicated by the tumor size, stage, and radiological feature. Both the timMRD-score and somatic mutations revealed consistent dynamic changes during perioperative periods. These results indicated that the timMRD model could reflect the abundance of tumor-specific differentially-methylated DNA fragments in peripheral blood.

A vast majority of the mutations detected from cfDNA are related to clonal hematopoiesis [22, 25]. To improve the accuracy of mutation profiling, we utilized a UMI-tagging technology, also referred to as duplex sequencing, to eliminate amplification errors and contaminants and improve the detection of somatic mutations with ultralow frequency [16, 17]. Moreover, the simultaneous profiling of leukocyte DNA enabled the removal of clonal hematopoietic mutations and ensured that variants reported were specific, tumor-derived mutations. The ubiquity of aberrant DNA methylation [26, 27] may contribute to the improved diagnostic sensitivity of a methylation-based assay more than the detection of a limited number of somatic mutations, particularly in early-stage cancer, which were also demonstrated by other published studies [9, 10, 17]. Since DNA methylation patterns are generally tissue-specific with distinct methylation profiles seen in blood and tumor cells [28], non-specific signals or technical noise can be eliminated in silico without requiring additional methylation data from paired leukocytes [29].

Blood-based detection of somatic mutations was highly specific in reflecting disease recurrence, particularly for patients with stage II/III disease and non-adenocarcinoma histology [3, 30, 31]. However, the sensitivity of mutation-based MRD analysis became limited in patients with low tumor burden or low tumor mutational burden, particularly in stage I, adenocarcinoma, or patients who are ctDNA-negative at baseline. Our study demonstrated the promising utility of tumor-informed methylation-based approach in disease monitoring at early postoperative time-points, including the patient subgroups with the above-mentioned low tumor burden. Multivariable regression analyses revealed a similar prognostic value between postoperative timMRD-score and TNM staging. Furthermore, timMRD-scores were also feasible for patients with stage I lung cancer and patients who were ctDNA-negative at baseline, which may help to accurately identify the population at higher risk of disease recurrence and guide their therapeutic and surveillance strategy before starting adjuvant chemotherapy approximately a month after surgery. Low-risk populations can maintain a moderate follow-up strategy; whereas high-risk populations may benefit from intensive follow-up and adjuvant therapy. In addition, during long-term follow-up, the high negative predictive value for timMRD-score indicates an accurate prediction of lower to no risk of relapse within a time frame for patients with low timMRD-scores. Patients with low timMRD-scores are basically not expected to experience relapse within four months and may not need to undergo radiologic imaging before four months. Overall, methylation-based disease monitoring may enable a personalized strategy in the postoperative management of lung cancer patients in line with the proposed TNM staging system [32, 33].

This study represents a proof-of-concept of a novel methylation-based cancer surveillance framework. Since the data generated by the timMRD model is dependent on the methylation markers included in the panel and the sequencing accuracy, our future plans include optimization of the probe range of the panel, the sequencing method, and the algorithm used for the model. Bisulfite-free methylation sequencing methods have demonstrated improved accuracy and cost-effectiveness, which is promising in liquid biopsy applications [34–36]. In view of the complementary advantages of mutation and methylation-based MRD detection, establishing a model that integrates both mutation and methylation may leverage the respective advantages of these two cfDNA-based approaches to further improve the accuracy of disease monitoring [37]. Furthermore, integrating other omics data, including fragmentomics and proteomics, may also enhance the accuracy in disease monitoring and warrants further exploration [4, 37–40].

There are limitations in our study. First, some data for the postoperative blood samples were not according to the set schedule or missing due to interruptions in regular patient follow-up caused by the coronavirus disease 2019 (COVID-19) pandemic. Second, the median follow-up was only 25 months for the MEDAL cohort, which is insufficient to monitor some patients with elevated postoperative timMRD-scores and identify disease-free patients with latent residual disease. However, about 80% of the relapsed patients from our cohort experienced disease relapse within two years [41] and the findings from the MEDAL cohort were successfully validated in the independent, non-overlapping cohort with a five-year follow-up data from the DYNAMIC study. Hence, we believe that our findings are conclusive and raise the potential clinical utility of methylation-based MRD detection. In the future, a multi-center study that includes a larger cohort and long-term follow-up is necessary to clarify these observations. In this study, timMRD provides a promising strategy for postoperative lung cancer surveillance, which could account for social economic benefits by reducing the combined costs from various post-operative screening approaches and ensure better adherence of the high-risk patients to intensive follow-ups and adjuvant therapy. With the development of technical innovations, reduced sequencing costs, and large-scale translational studies, we anticipate the adapting sensitive, cost-effective and individualized methylation-based MRD detection tests from the bench-side to the bedside will eventually benefit the general public as the technology evolves.

## Conclusions

In this study, the dynamic characteristics of cancer-related methylation signals was explored using personalized tumor-informed methylation-based MRD (timMRD). In the MEDAL cohort, the pre-operative timMRD scores showed a positive correlation with tumor burden, invasiveness, and the presence and abundance of somatic mutations. Furthermore, the postoperative timMRD score emerged as an independent prognostic factor for lung cancer. Notably, the timMRD scores outperformed tumor-informed fixed panel in identifying relapsed patients during postoperative follow-up, even among patients with lower tumor burden. This study has provided clinical evidence on the feasibility of tumor-informed methylation-based MRD detection, contributing an incremental step in developing effective postoperative cancer surveillance strategies in early-stage lung cancer.

## Supplementary Information

**Additional file 1.** Supplementary Methods.

**Additional file 2:** **Figure S1.** Detailed workflow of the methylation-based analysis and timMRD model. **Figure S2.** Quality control of bisulfite sequencing for all patient tissue and blood samples. **Figure S3.** Distinct methylation profile of tumor tissues. **Figure S4.** Somatic mutation profile of baseline blood samples for 155 patients with paired tissue-based mutation data. **Figure S5.** Spike-in dilution experiments and numerical simulation trials demonstrate accuracy and robustness of the timMRD model. **Figure S6.** Relationship between tumor-informed ctDNA mutation, methylation status, and clinical features. **Figure S7.** Perioperative dynamics of timMRD scores, MethylMean, and tumor-informed ctDNA somatic mutation status. **Figure S8.** Perioperative management does not affect timMRD scores. **Figure S9.** TimMRD scores are positively correlated with somatic mutations (maxAF) in all ctDNA-positive samples. **Figure S10.** Plasma B and Plasma C reflect disease relapse using timMRD scores and tumor-informed ctDNA somatic mutations at postoperative time-points, but not MethylMean. **Figure S11.** Prognostication using ctDNA mutation status or timMRD score evaluated at postoperative follow-up time-points for either Plasma B or Plasma C in MEDAL cohort. **Figure S12.** Two-year prognostication using timMRD score evaluated at postoperative follow-up time-points using Plasma B or Plasma C in MEDAL cohort. **Figure S13.** Dynamics of ctDNA mutation and timMRD score in two relapsed patients of the MEDAL cohort.

**Additional file 3: Table S1.** Baseline clinicopathologic characteristics of the 36 patients in the DYNAMIC cohort. **Table S2.** Summary of data in related studies on ctDNA-based MRD detection in early-stage lung cancer. **Table S3.** Analytical performance of timMRD model at various cutoff levels (percentile of chi-square distribution for timMRD scores) and tumor-informed ctDNA mutation status for the 155 patients with tumor-informed ctDNA mutation data at the last follow-up. Of the 155 patients, 11 patients had follow-up data at 120 days before relapse and 52 patients had follow-up data at the same time-point.

**Additional file 4: Data S1.** Detailed clinical information of the MEDAL cohort.

**Additional file 5: Data S2.** Detailed molecular information of the 155 patients evaluable for tumor-informed ctDNA mutation status from the MEDAL cohort.

**Additional file 6: Data S3.** Raw methylation scores for tissue samples of the MEDAL cohort.

**Additional file 7: Data S4.** Raw methylation scores for blood samples of the MEDAL cohort.

## Data Availability

The clinical, genomic, and epigenomic datasets analyzed for this study are summarized as Additional file 4: Data S1, Additional file 5: Data S2, Additional file 6: Data S3 and Additional file 7: Data S4. The raw files for targeted genomic and targeted bisulfite sequencing are available in the National Omics Data Encyclopedia (NODE), hosted by the Big Data Center (BMDC) available as accession number (OEP003150) and can be accessed thru the URL (https://www.biosino.org/node/project/detail/OEP003150) after reasonable request. Codes and scripts developed for this study are available at https://github.com/bnr-ed/timMRD.

## Abbreviations

AUC: Area under the curve
cfDNA: Circulating cell-free DNA
CI: Confidence intervals
ctDNA: Circulating tumor DNA
DMB: Differentially methylated blocks
HR: Hazard ratio
MEDAL: MEthylation based Dynamic Analysis for Lung cancer
MethylMean: Mean methylation level
MRD: Minimal residual disease
NSCLC: Non-small-cell lung cancer
timMRD: Tumor-informed methylation-based MRD
