# Supplementary material for: Individualized dynamic methylation-based analysis of cell-free DNA in postoperative monitoring of lung cancer
Source: BMC Med. 2023 Jul 14;21:255. doi: 10.1186/s12916-023-02954-z (PMC10349423; doi:10.1186/s12916-023-02954-z)
Supplement: Supplementary file 1 — Additional file 1. Supplementary Methods. [file 12916_2023_2954_MOESM1_ESM.docx]

**Supplementary Methods**

***Patient selection criteria***

Patients at the Department of Thoracic Surgery of the Peking University People’s Hospital were prospectively enrolled between August 2018 and July 2019. According to the published protocol for this study (1), all eligible patients were candidates for curative surgery, with a suspected clinical stage between IA and IIIA based on pre-operative examination. The exclusion criteria were having: (1) pure ground-glass opacity; (2) received neoadjuvant therapy; (3) a history of malignancy within five years; (4) multifocal lung cancer and complete resection is unfeasible; (5) a pathology other than NSCLC; (6) a tumor pathologically-staged as IIIB-N3 or IV; (7) unqualified or failure to obtain blood or tissue samples; (8) refused or withdrawn consent; and (9) other circumstances deemed inappropriate for this study, including blood transfusion, gestation, and others.

***DNA isolation***

Genomic DNA and cell-free DNA (cfDNA) were respectively purified from flash-frozen tumor samples and plasma samples using a QIAamp DNA tissue kit and QIAamp circulating nucleic acid kit, according to the manufacturer’s standard protocol (Qiagen, Hilden, Germany). DNA isolated from tissue and plasma samples were quantified using the Qubit 3.0 fluorometer with dsDNA high sensitivity assay kit (Life Technologies, Carlsbad, CA, USA).

***Unique molecular identifier (UMI)-based targeted ultra-deep mutation sequencing***

As described previously (2, 3), acoustically sheared tissue DNA and cfDNA fragments between 200 and 400 bp were purified, end-repaired, and A-tailed. cfDNA were ligated with UMI-containing sequencing adapters designed by Burning Rock Biotech. Tissue DNA were processed accordingly with non-UMI sequencing adapters. After purification using Agencourt AMPure XP Kit (Beckman Coulter, CA, USA), the adapter-ligated DNA were hybridized with capture probes baits, hybrid-selected with magnetic beads, and amplified. Target capture was performed using a commercial panel with RNA baits designed for 168 lung cancer-related genes, spanning 273 kilobases of the human genome (Lung Plasma UMI; Burning Rock Biotech, Guangzhou, China), which was validated in a report by the FDA-led sequencing quality control phase 2 (SEQC2) oncopanel sequencing working group (4). The indexed samples were sequenced on an Illumina NovaSeq 6000 (Illumina, San Diego, CA, USA) with 2 × 150 bp and target sequencing depth of 1,000× for tissue samples and 30,000× for plasma samples. Sequencing data were analyzed using proprietary computational algorithms optimized for somatic variant calling.

***Bisulfite targeted sequencing and sequencing data analysis***

The library preparation for bisulfite targeted sequencing was performed following the optimized protocols for ELSA-seq as described previously (3, 5). Briefly, the purified gDNA and cfDNA were treated with sodium bisulfite (EZ-96 DNA Methylation-Lightning MagPrep, Zymo Research, Orange, CA, USA) to convert all the cytosine residues to uracil, but leaving all the 5-methylcytosine residues unchanged. Subsequently, the converted single-stranded DNA were ligated to a splinted adapter, amplified with uracil-tolerating DNA polymerase and extension primers, ligated with adaptors, and again amplified to obtain the whole-genome bisulfite sequencing library. Target enrichment was performed using custom-designed lung cancer methylation profiling RNA baits covering 80,672 CpG sites that span 1.05 MBs of the human genome. As described previously (3), the 80,672 CpG sites included in the targeted bisulfite panel were selected by analyzing lung cancer-related methylation markers using the Illumina Infinium HumanMethylation450k microarray-derived methylome dataset for lung tumor and normal tissue samples from The Cancer Genome Atlas ([https://www.cancer.gov/tcga](https://www.cancer.gov/about-nci/organization/ccg/research/structural-genomics/tcga)). After the hybridization step, biotinylated RNA probe-bound library fragments were selectively enriched and amplified. The target libraries were finally quantified by real-time PCR (Kapa Biosciences, Wilmington, MA, USA) and sequenced on NovaSeq 6000 (Illumina, San Diego, CA, USA) using 2 × 150 bp cycles. Bisulfite sequencing data were analyzed using an optimized pipeline. Custom adaptor sequences and low-quality bases were removed by trimmomatic (version 0.32). BWA-meth (version 0.2.2) was used to align paired-end reads to CtoT- and GtoA-transformed hg19 genome (6). After alignment, PCR duplicates were marked by Samblaster (version 0.1.20) (7). The reads with either low mapping quality (MAPQ <20) or improper pairing were removed by Sambamba (version 0.4.7) (8) from further downstream analyses. Paired-read sequences were merged using in-house scripts by clipping overlapping reads to avoid double-counting of methylation calls. The analytical validation study of the ELSA-seq has been published (3).

***Healthy cohort***

Blood samples from an in-house cohort of 312 healthy donors were used as the normal cfDNA reference for setting the baseline for timMRD model construction. The healthy cohort was comprised of 82.1% (n = 256) males, 13.8% (n = 43) females, and 4.2% (n = 13) data unavailable. The median age of the cohort was 33.0 years, ranging between 16-89 years. The inclusion of a cohort with a wider age range ensured that methylation changes associated with age are accounted for. The healthy cohort had no history and/or family history of cancer, had normal serum levels of cancer biomarkers, and were not detected with lung cancer using low-dose computed tomography (LDCT).

***Stratifying differentially methylated blocks (DMBs)***

To define the association of methylation patterns among the CpG sites covered by the targeted bisulfite sequencing panel, a region-splitting algorithm was used to calculate the distance between the CpG sites and correlate the methylation levels in these sites to obtain their similarity. As a result, a total of 8,312 methylation blocks (MBs), defined as the genomic region covering multiple neighboring CpG sites, were generated based on the algorithm. Among these, 84% of the blocks were annotated in genes, wherein 59% were located in promoter regions, 7% in exons, and 18% in introns. The remaining 16% were annotated in intergenic regions. Based on the high similarity of CpG sites in each block, the blocks were regarded as biomarkers for further analysis. The cancer-related DMBs were identified from the resected lung tumor samples, tumor-adjacent normal tissue samples, and blood samples obtained from 195 patients. The tumor-adjacent normal tissues were confirmed to have a tumor cell percentage of <5% by histological examination, while the lung tumor samples were histologically confirmed to have ≥30% tumor cells. DMBs that were >20% methylated or unmethylated in tumor samples relative to the matched tumor-adjacent normal samples were considered as hypermethylated or hypomethylated, respectively. Blood samples from healthy individuals (demographics described in the subsection on Healthy cohort) were used as the normal cfDNA reference to represent the non-tumor fraction of cfDNA. The comparison of methylation signals between the normal cfDNA reference and both tumor tissue and tumor-adjacent normal tissue samples from the patient enables identification of tissue-specific DMBs and aids in amplifying the methylation signals in test plasma samples, particularly at low cfDNA abundance. The DMBs were defined as follows: In the $j$-th methylation block, the methylation level $\beta_{ij}$ for the $i$-th sample was calculated as $\beta_{ij}={M_{ij}}/{N_{ij}}$, wherein $M_{ij}$ represents the number of methylated CpG sites per *j-*th methylation block for *i-*th sample and $N_{ij}$ represents the total number of methylated (M) and unmethylated (U) CpG sites or the total methylation score per *j-*th methylation block for *i-*th sample. Using a two-sided paired t-test for the $\beta_{ij}$ methylation levels in 195 matched tumor-adjacent normal samples, we identified 3,159 DMBs with p-values below the Benjamini-Hochberg corrected threshold of 0.05. Personalized DMBs were further selected from these 3,159 MBs based on the individual $\beta_{ij}$ values. The mean methylation level (MethylMean) per sample was obtained by averaging the methylation level $\beta_{ij}$ of all DMBs detected in that sample.

***Evaluation of model accuracy using spike-in standards***

The quantitative accuracy of timMRD model was evaluated using tumor cell line spike-in experiments. H2209, a lung cancer cell standard, was serially diluted across a range of tumor fractions using GM24385, a normal B-lymphocyte cell standard. Targeted bisulfite sequencing was performed on DNA isolated from serially diluted preparations. Methylation levels were analyzed using the timMRD model. The results of these dilution experiments were presented in Figure S5A.

***Numerical simulation trials***

Two numerical simulation studies were performed to validate the accuracy of the timMRD model. First, we evaluated whether the timMRD score can reflect the quantity of tumor-derived DNA. The proportion of circulating tumor DNA was reflected as simulated cfDNA tumor fraction percentage (referred to as $\alpha_{i}$). Using single-parameter simulation, simulated cfDNA tumor fraction (%) was randomly chosen from the list {0, 0.01, 0.03, 0.1, 0.3, 1, 3, 10}, while the proportion of normal lung tissues, reflected as simulated normal fraction percentage (referred to as $\gamma_{i}$) was set at 0%. Then the methylation counts {$M_{ij},N_{ij}$} were generated from the beta-binomial distribution. Given $\gamma_{i}=0$, the results for MLE $\hat{\alpha_{i}}$ were presented in Figure S5B. We then evaluated whether timMRD score would be affected by DNA derived from the normal lung tissues using paired-parameter simulation. For this part, each parameter $\alpha_{i}$ and $\gamma_{i}$ were randomly selected from {0, 0.1, 1, 10}. We generated 100 replications of {$M_{ij},N_{ij}$} for each fixed parameter combination {$\alpha_{i},\gamma_{i},{\beta_{ij}}^{\left( T \right)},{\beta_{ij}}^{(N)},p_{j}, q_{j}$}. We then applied MLE algorithm to calculate $\hat{\alpha i}$ and $\hat{\gamma i}$ given the parameters {${\beta_{ij}}^{\left( T \right)},{\beta_{ij}}^{(N)},p_{j}, q_{j}$} with the results presented in Figure S5C.

***Definition of radiological and pathological risk of recurrence***

Several radiological and pathological features can reflect the invasiveness and prognosis of NSCLC. Pulmonary nodules can be categorized radiologically as solid, pure ground-glass opacity (GGO), or a mixture of solid and GGO (mixed). Increased invasiveness has been observed among radiologically solid nodules than those with GGO components (9). Tumors with solid- or micropapillary-predominant adenocarcinoma histology showed higher invasiveness than that of lepidic- and acinar-predominant adenocarcinoma (10). For this particular analysis, pathological low-risk was defined by the following three criteria: 1. having adenocarcinoma with lepidic- and acinar-predominant histology; 2. the absence of solid and micropapillary component; and 3. the absence of intravascular tumor thrombus, visceral pleural invasion, and lymph node metastasis. Cases that did not meet these criteria were categorized as high-risk.

***Definition of radiological and pathological risk of recurrence***

Several radiological and pathological features can reflect the invasiveness and prognosis of NSCLC. Pulmonary nodules can be categorized radiologically as solid, pure ground-glass opacity, or a mixture of solid and GGO (mix). Increased invasiveness has been observed among radiologically solid nodules than those with GGO components (9). Tumors with solid- or micropapillary-predominant adenocarcinoma histology showed higher invasiveness than that of lepidic- and acinar-predominant adenocarcinoma (10). For this particular analysis, pathological low-risk was defined by the following criteria: adenocarcinoma with lepidic- and acinar-predominant predominant histology, with the absence of solid and micropapillary component; and the absence of intravascular tumor thrombus, visceral pleural invasion, and lymph node metastasis. Cases that did not meet these criteria were categorized as high-risk.

***Statistical analysis and data visualization***

Figures illustrating our data were prepared using R statistical programming (R version 3.4.0) by implementing the following packages: ggplot2 (version 3.3.5), ComplexHeatmap (version 2.6.2), survival (version 3.2-13), survminer (version 0.4.9), ggpubr (version 0.4.0), and ggsci (version 2.9).

**References**

1. Kang G, Chen K, Yang F, Chuai S, Zhao H, Zhang K, et al. Monitoring of circulating tumor DNA and its aberrant methylation in the surveillance of surgical lung Cancer patients: protocol for a prospective observational study. BMC Cancer. 2019;19(1).

2. Yang Y, Zheng D, Wu C, Lizaso A, Ye J, Chuai S, et al. Detecting Ultralow Frequency Mutation in Circulating Cell‐Free DNA of Early‐Stage Nonsmall Cell Lung Cancer Patients with Unique Molecular Identifiers. Small Methods. 2019;3(10):1900206.

3. Liang N, Li B, Jia Z, Wang C, Wu P, Zheng T, et al. Ultrasensitive detection of circulating tumour DNA via deep methylation sequencing aided by machine learning. Nat Biomed Eng. 2021;5(6):586-99.

4. Deveson IW, Gong B, Lai K, LoCoco JS, Richmond TA, Schageman J, et al. Evaluating the analytical validity of circulating tumor DNA sequencing assays for precision oncology. Nat Biotechnol. 2021;39(9):1115-28.

5. Kang G, Chen K, Yang F, Chuai S, Zhao H, Zhang K, et al. Monitoring of circulating tumor DNA and its aberrant methylation in the surveillance of surgical lung Cancer patients: protocol for a prospective observational study. BMC Cancer. 2019;19(1):579.

6. Pedersen BS, Eyring K, De S, Yang IV, Schwartz DA. Fast and accurate alignment of long bisulfite-seq reads. arXiv:14011129v2 [q-bioGN]. 2014.

7. Faust GG, Hall IM. SAMBLASTER: fast duplicate marking and structural variant read extraction. Bioinformatics. 2014;30(17):2503-5.

8. Tarasov A, Vilella AJ, Cuppen E, Nijman IJ, Prins P. Sambamba: fast processing of NGS alignment formats. Bioinformatics. 2015;31(12):2032-4.

9. Naidich DP, Bankier AA, MacMahon H, Schaefer-Prokop CM, Pistolesi M, Goo JM, et al. Recommendations for the management of subsolid pulmonary nodules detected at CT: a statement from the Fleischner Society. Radiology. 2013;266(1):304-17.

10. Travis WD, Asamura H, Bankier AA, Beasley MB, Detterbeck F, Flieder DB, et al. The IASLC Lung Cancer Staging Project: Proposals for Coding T Categories for Subsolid Nodules and Assessment of Tumor Size in Part-Solid Tumors in the Forthcoming Eighth Edition of the TNM Classification of Lung Cancer. Journal of thoracic oncology : official publication of the International Association for the Study of Lung Cancer. 2016;11(8):1204-23.
