# Supplementary material for: Individualized dynamic methylation-based analysis of cell-free DNA in postoperative monitoring of lung cancer
Source: BMC Med. 2023 Jul 14;21:255. doi: 10.1186/s12916-023-02954-z (PMC10349423; doi:10.1186/s12916-023-02954-z)
Supplement: Supplementary file 2 — Additional file 2: Figure S1. Detailed workflow of the methylation-based analysis and timMRD model. Figure S2. Quality control of bisulfite sequencing for all patient tissue and blood samples. Figure S3. Distinct methylation profile of tumor tissues. Figure S4. Somatic mutation profile of baseline blood samples for 155 patients with paired tissue-based mutation data. Figure S5. Spike-in dilution experiments and numerical simulation trials demonstrate accuracy and robustness of the timMRD model. Figure S6. Relationship between tumor-informed ctDNA mutation, methylation status, and clinical features. Figure S7. Perioperative dynamics of timMRD scores, MethylMean, and tumor-informed ctDNA somatic mutation status. Figure S8. Perioperative management does not affect timMRD scores. Figure S9. TimMRD scores are positively correlated with somatic mutations (maxAF) in all ctDNA-positive samples. Figure S10. Plasma B and Plasma C reflect disease relapse using timMRD scores and tumor-informed ctDNA somatic mutations at postoperative time-points, but not MethylMean. Figure S11. Prognostication using ctDNA mutation status or timMRD score evaluated at postoperative follow-up time-points for either Plasma B or Plasma C in MEDAL cohort. Figure S12. Two-year prognostication using timMRD score evaluated at postoperative follow-up time-points using Plasma B or Plasma C in MEDAL cohort. Figure S13. Dynamics of ctDNA mutation and timMRD score in two relapsed patients of the MEDAL cohort. [file 12916_2023_2954_MOESM2_ESM.pdf]

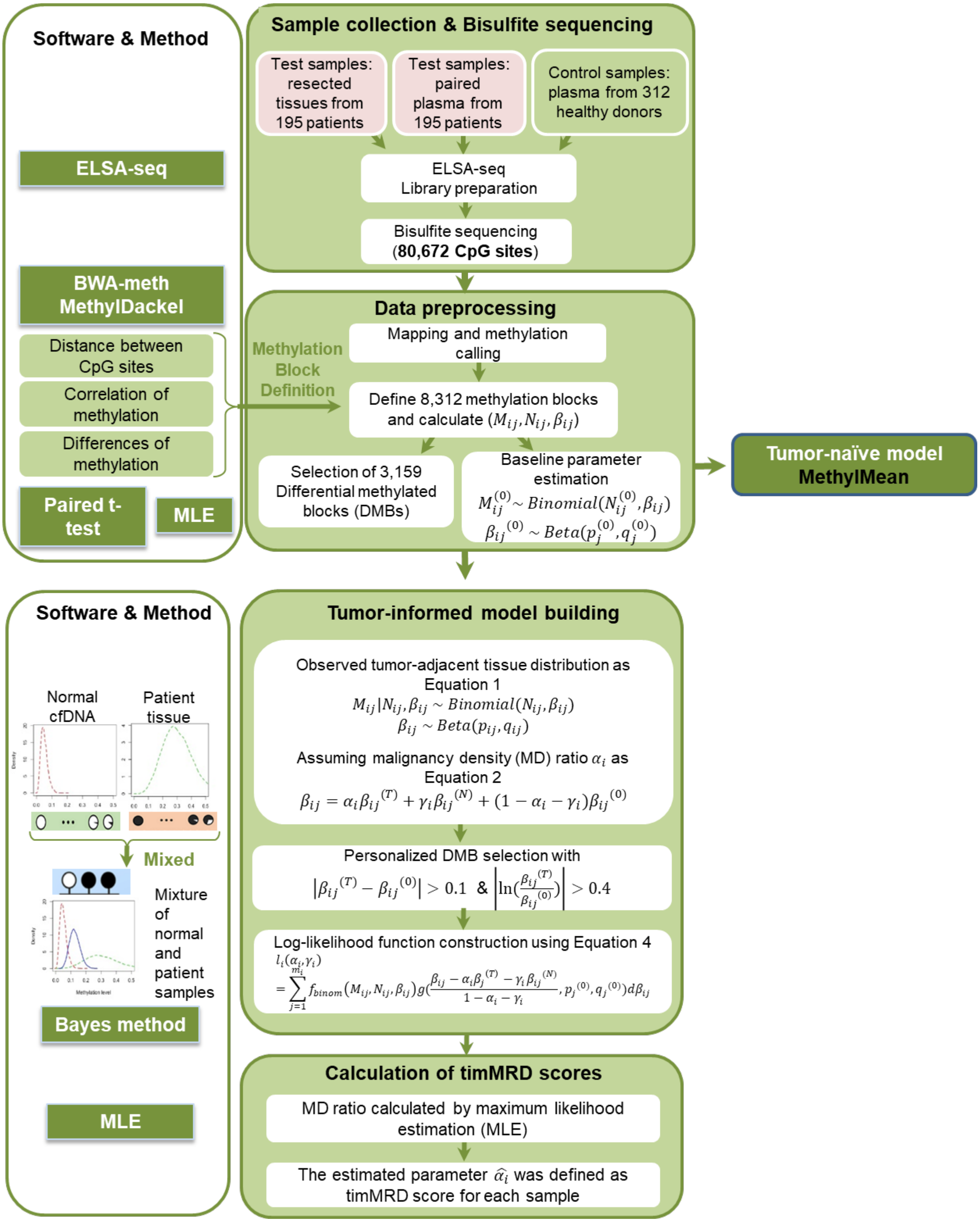

Figure S1. Detailed workflow of the methylation-based analysis and timMRD model. Flow diagram illustrating the steps involved in methylation sequencing data analysis, stratification of differentially methylated blocks, and constructing the timMRD statistical model (See Supplementary Methods). Related to Figure 1B.

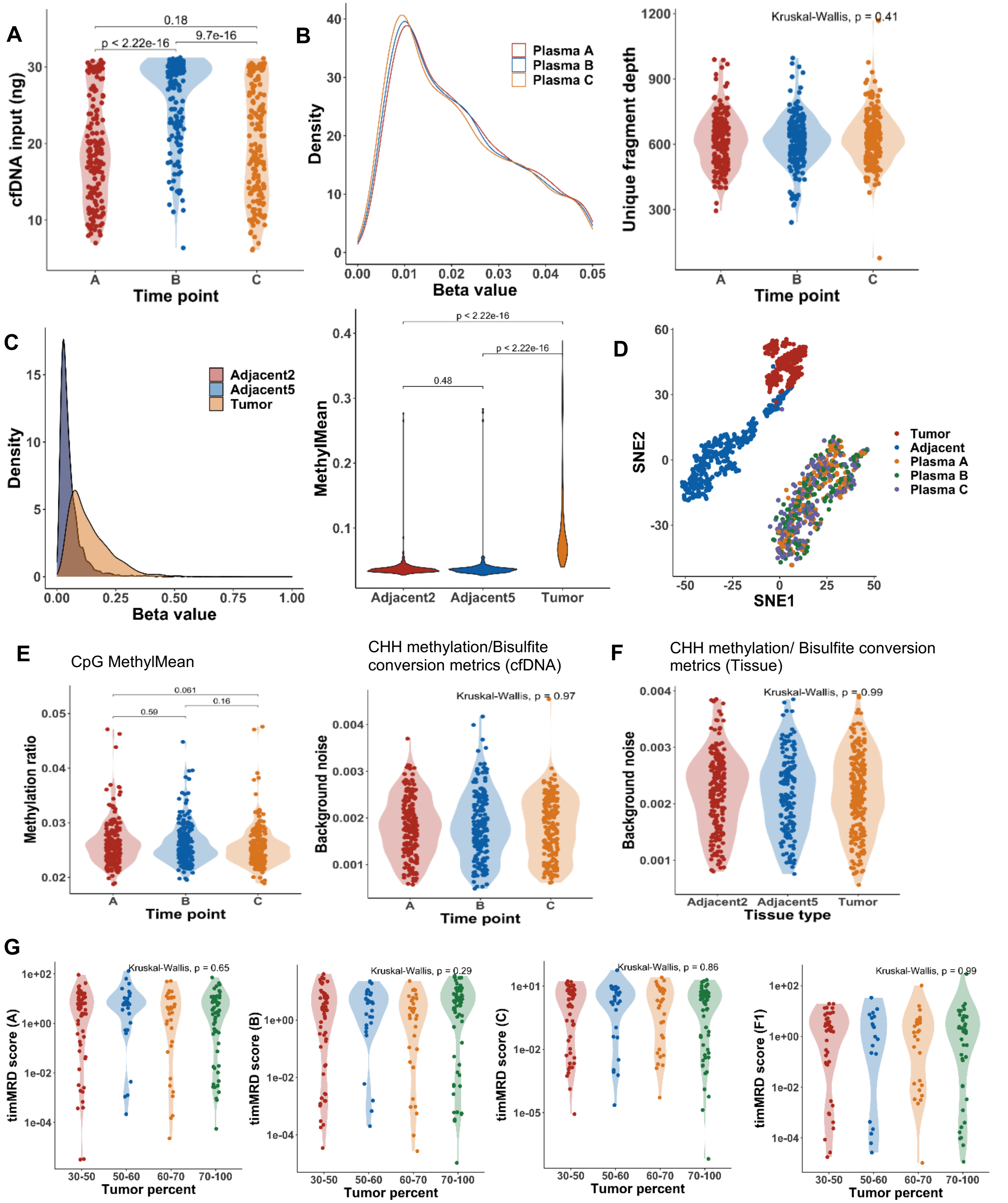

Figure S2. Quality control of bisulfite sequencing for all patient tissue and blood samples. A. Cell-free DNA (cfDNA) input for bisulfite sequencing for all patient blood samples in nanograms (ng). **B**. The distribution of average methylation levels of each CpG block per sample (Beta value or β_{ij}) (**B-left panel, C**), unique fragment depth (**B-right panel**), and average methylation levels of all CpG blocks or the average Beta value per sample (MethylMean) (**E-left panel**) for each of the blood samples at various time points (**B, E**) and tissue samples (**D, F**). **E-F**. Bisulfite conversion metrics for non-CpG sites, also referred to as MethylMean of CHH sites (wherein H represents nucleotides C, A, or T), of all patient plasma samples at perioperative time points and tissue samples, including normal tissue samples obtained 2 cm (Adjacent2) or 5 cm (Adjacent5) away from the tumor margin and tumor tissue samples. **G**. Relationship between tumor cell percentage of tumor tissue samples and timMRD-scores at different time-points. Kruskal-Wallis H test was performed to compare the difference between groups. Statistical significance was defined as P-value < 0.05.

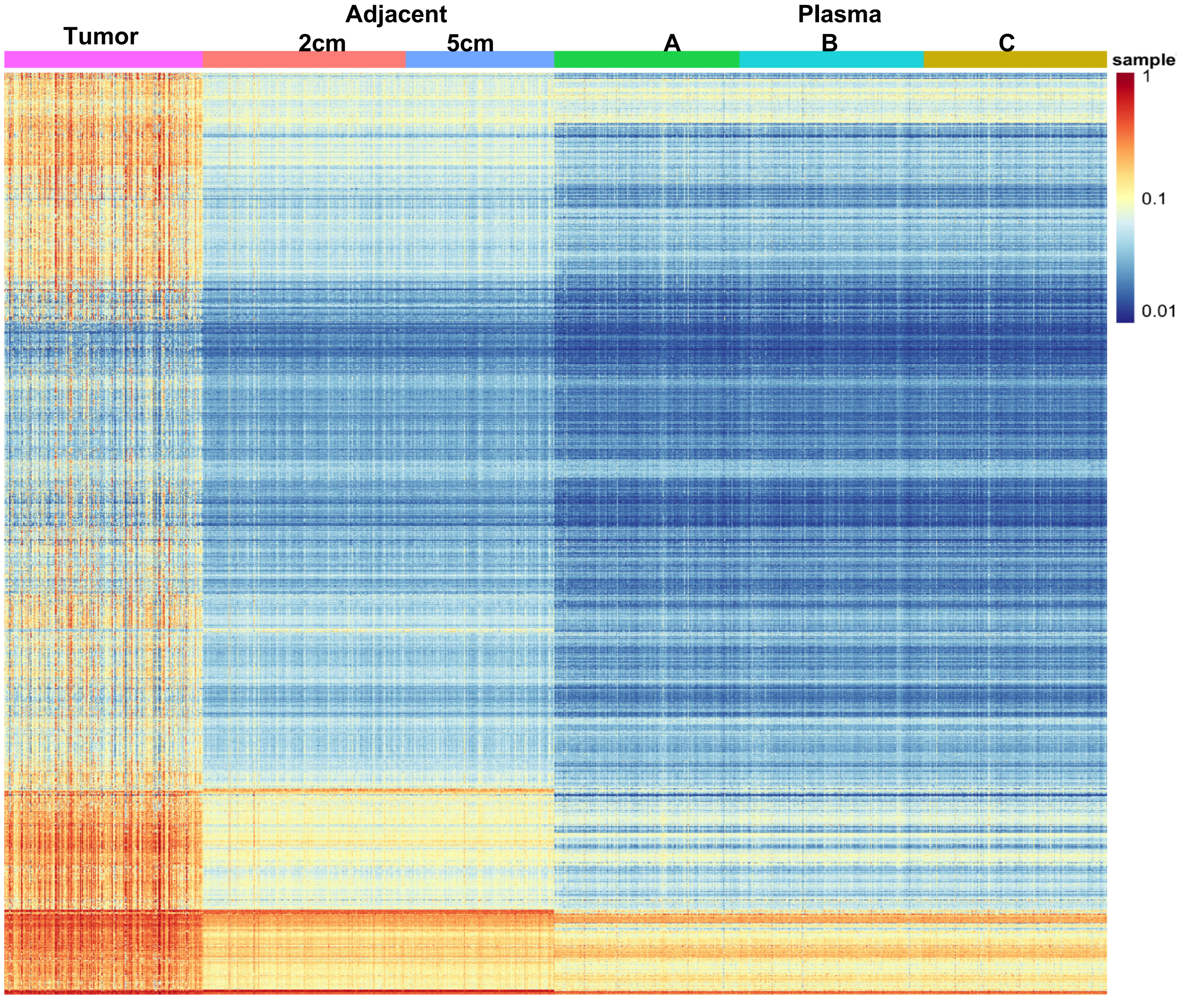

Figure S3. Distinct methylation profile of tumor tissues. Unsupervised hierarchical clustering of the hypermethylation ratios of tumor tissues, tumor-adjacent normal tissues obtained 2cm or 5cm away from the tumor margin, and plasma samples at perioperative time-points. Related to Figure 2A.

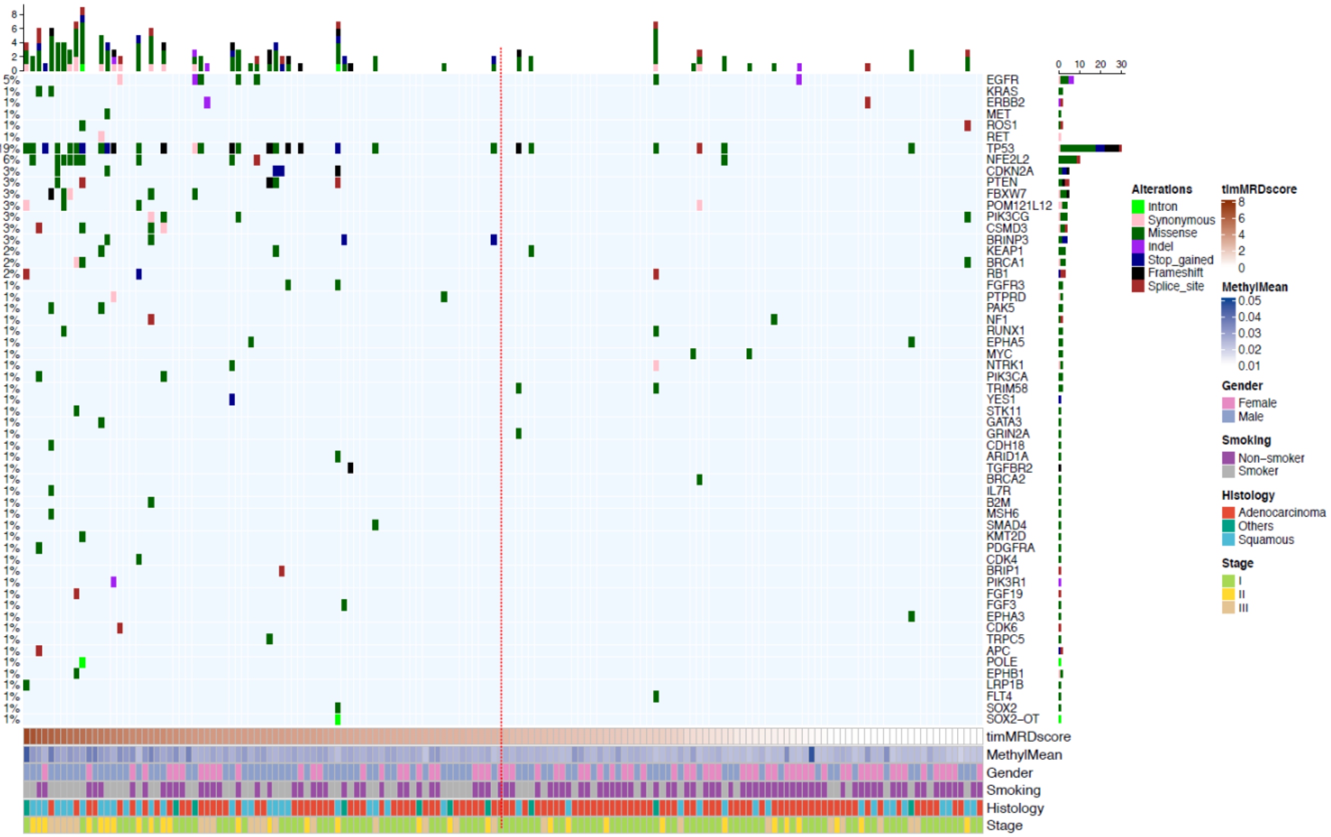

Figure S4. Somatic mutation profile of baseline blood samples for 155 patients with paired tissue-based mutation data. Each column represents a patient and each row represents a gene. The data were arranged according to descending order of timMRD scores. The colored bars located at the bottom of the OncoPrint correspond to the timMRD scores, MethylMean, gender, smoking status, histology, and pathological stage of each patient. Genes are indicated on the right of the OncoPrint, while mutation rates are indicated on the left. Component bar plots at the top represent the overall number of mutations per patient. Mutation types are denoted by colors.

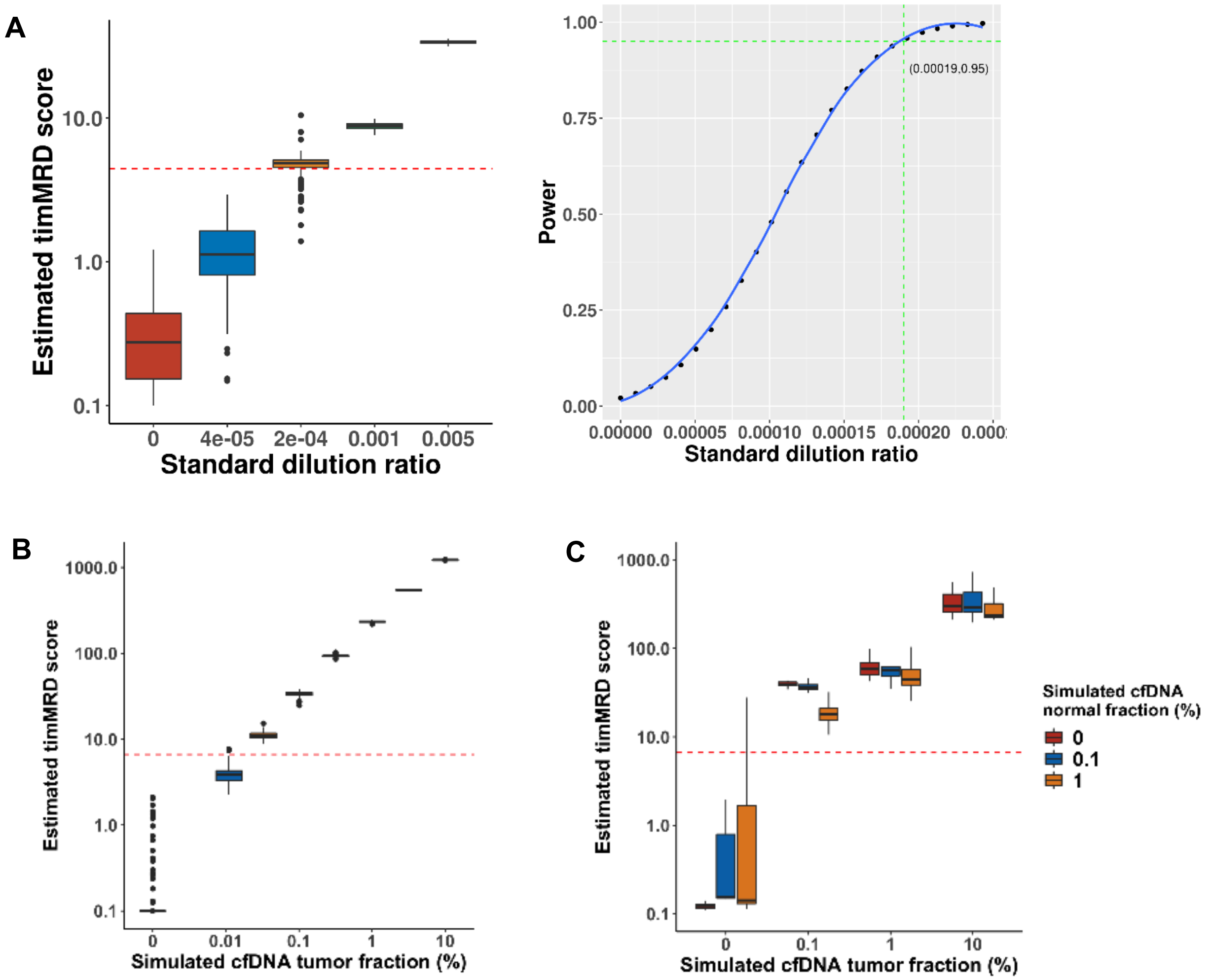

Figure S5. Spike-in dilution experiments and numerical simulation trials demonstrate accuracy and robustness of the timMRD model. A. The accuracy of timMRD model was assayed *in vitro* using serially diluted spike-in standard/reference samples as described in the Supplementary Methods. Red dotted line indicates the timMRD-score cutoff of 5.412. The green dotted line on the right panel indicates the intersection between the tumor fraction (0.0002) and power of 0.95, suggesting that timMRD model could achieve 95% accuracy in tumor fraction as low as 0.0002 and also indicates the limit of detection of the timMRD model. **B-C.** Single-parameter (**B**) and paired-parameter (**C**) numerical simulation trials demonstrate the precision and robustness of the timMRD model. Red dotted lines indicate timMRD-score cutoff of 5.412 (See Supplementary Methods for details).

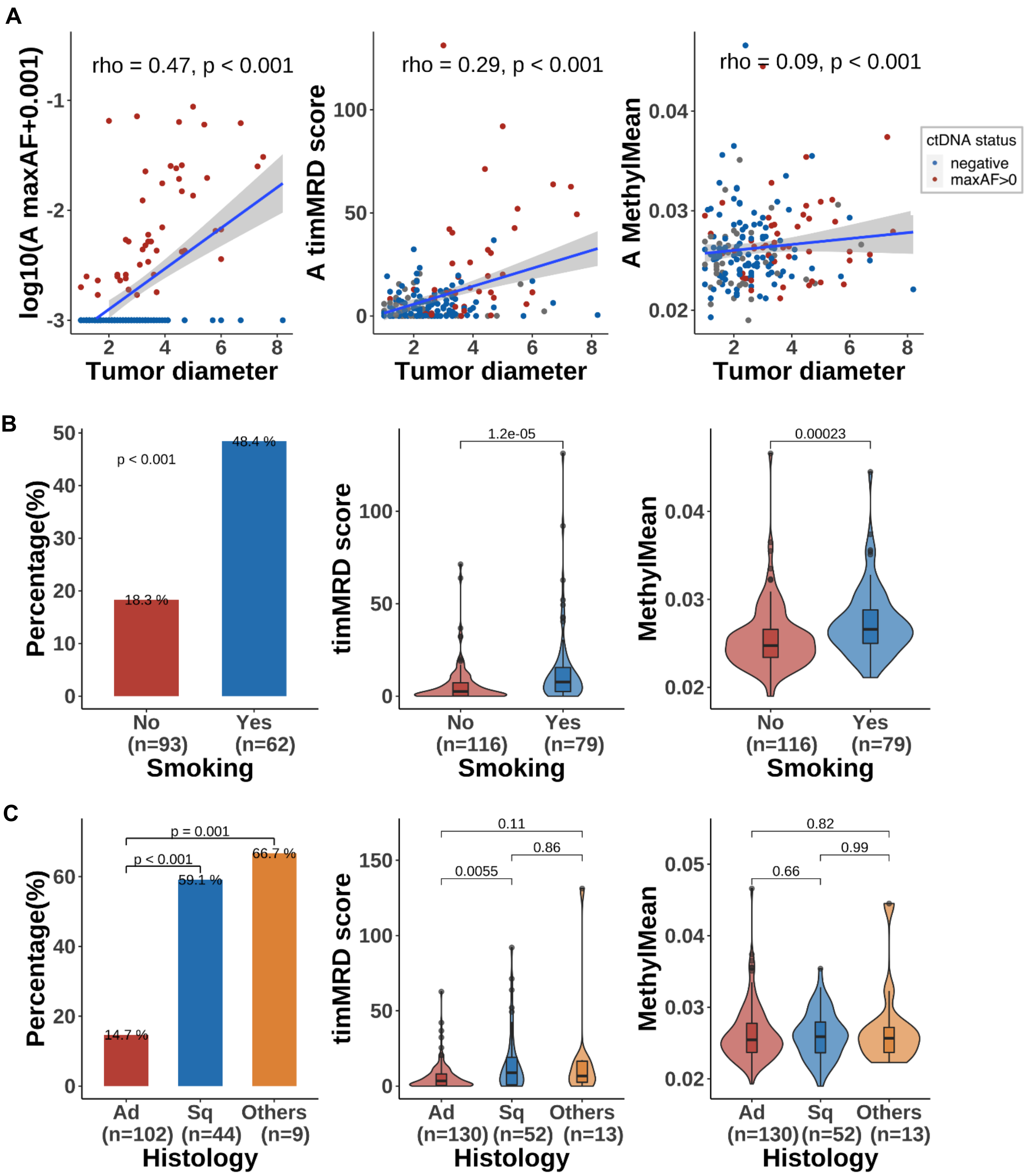

Figure S6. Relationship between tumor-informed ctDNA mutation, methylation status, and clinical features. A. Relationship between tumor diameter and MethylMean (left), timMRD score (middle), and maxAF of ctDNA mutations (right). Blue line denotes the best-fitting line. Gray shadow denotes 95% confidence intervals. Spearman rank-order correlation test was performed to analyze the correlation between the molecular assay and clinical features. **B-C**. Bar graphs illustrating the tumor-informed ctDNA mutation-positive rates (left), and violin plots illustrating the timMRD scores (middle), and MethylMean (right) according to smoking status (**B**), and histological subtypes (**C**). Pairwise comparisons were performed using Fisher's exact test or Wilcoxon signed-rank test. Statistical significance was defined as P-value <0.05.

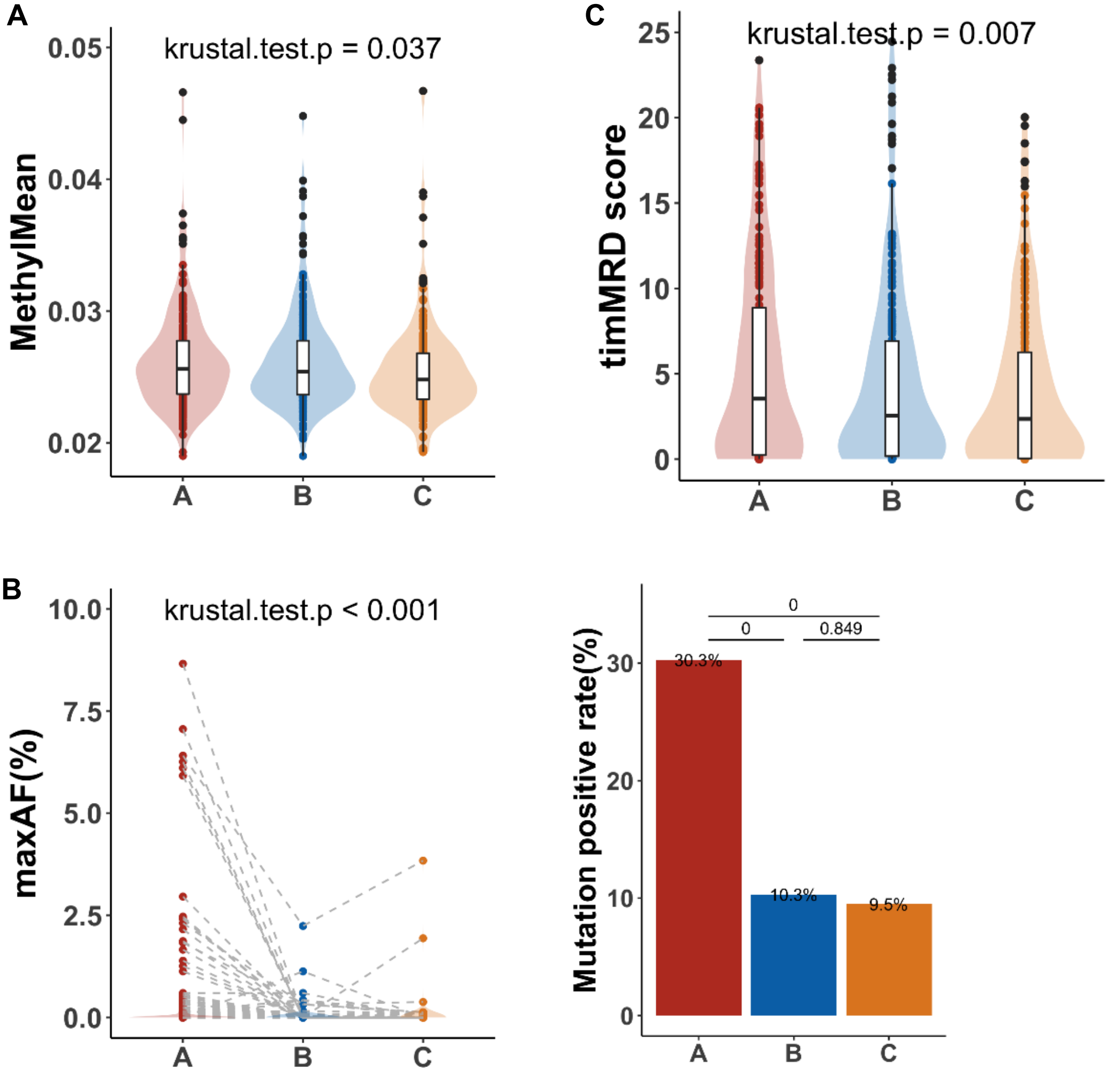

Figure S7. Perioperative dynamics of timMRD scores, MethylMean, and tumor-informed ctDNA somatic mutation status. A-C. Plots illustrating the dynamic changes in MethylMean (**A**), maxAF and tumor-informed ctDNA mutation detection rate (**B**), and timMRD scores (**C**) at perioperative time points for Plasma A, Plasma B, and Plasma C. Kruskal-Wallis H test was performed to compare the difference across the groups. Statistical significance was defined as P-value < 0.05.

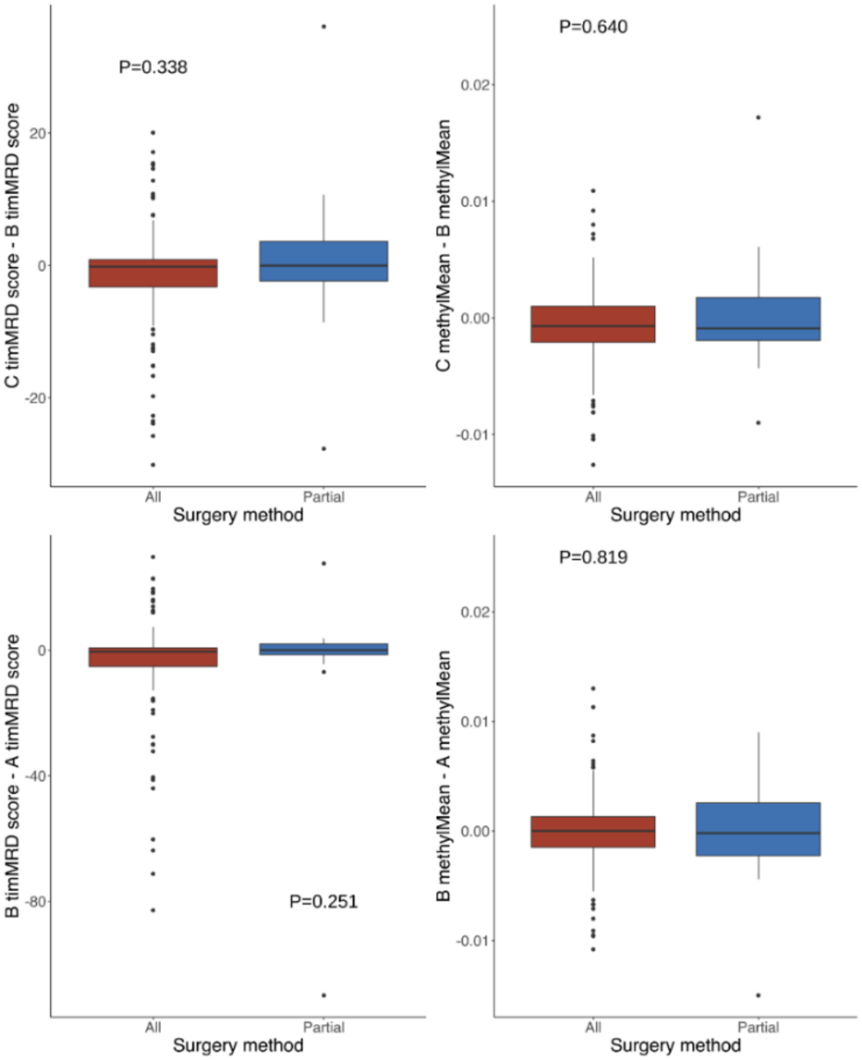

Figure S8. Perioperative management does not affect timMRD scores. Variations in methylation status have no statistical difference with the scope of the surgery. All (red bars) and partial (blue bars) denote lobectomy and wedge resection, respectively. Kruskal-Wallis H test was performed to compare the difference between groups. Statistical significance was defined as P-value < 0.05.

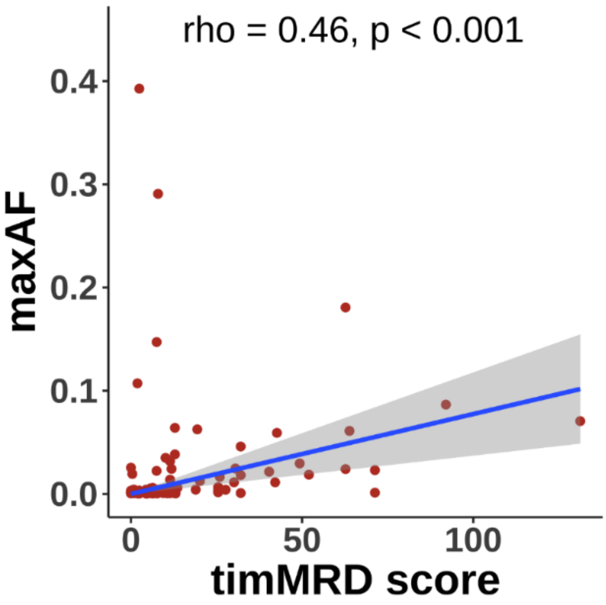

Figure S9. TimMRD scores are positively correlated with somatic mutations (maxAF) in all ctDNA-positive samples (n=47). Blue line denotes the best-fitting line. Gray shadow indicates 95% confidence intervals. Pearson correlation test was performed to analyze the correlation between the timMRD sore and maxAF of ctDNA somatic mutations.

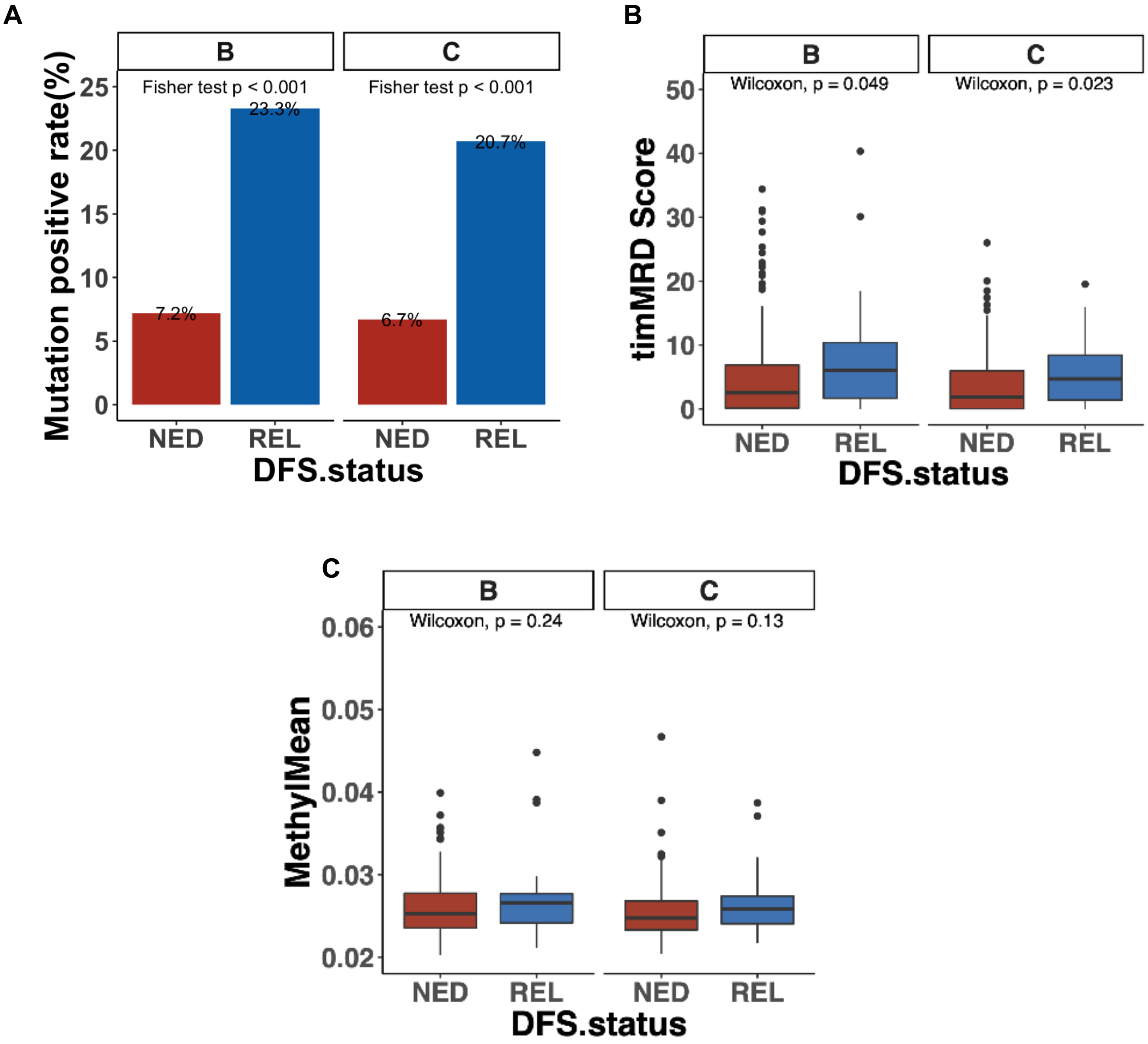

Figure S10. Plasma B and Plasma C reflect disease relapse using timMRD scores and tumor-informed ctDNA somatic mutations at postoperative time-points, but not MethylMean. Comparison of ctDNA mutation positive rates (**A**), timMRD scores (**B**), and MethylMean (**C**) for Plasma B and Plasma C between patients with no evidence of disease (NED) and patients who experienced disease relapse (REL). Fisher test or Wilcoxon signed-rank test was performed to compare the difference between groups as indicated. Statistical significance was defined as P-value <0.05.

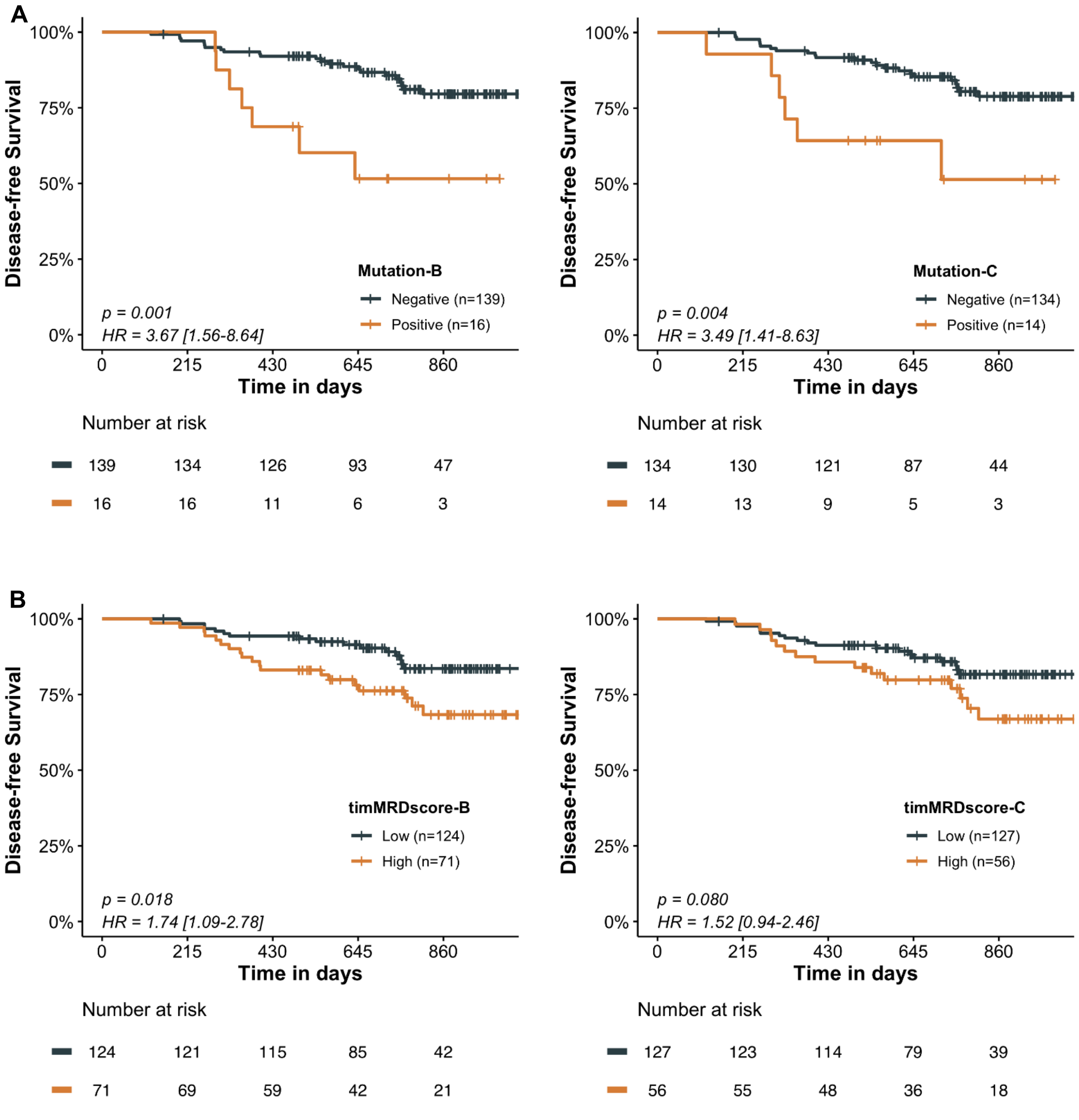

Figure S11. **Prognostication using ctDNA mutation status or timMRD score evaluated at postoperative follow-up time-points for either Plasma B or Plasma C in MEDAL cohort.** Kaplan-Meier analysis of disease-free survival (DFS, expressed in days) according to ctDNA mutation status (**A**) and timMRD scores (**B**) for either Plasma B or Plasma C of the MEDAL cohort with tumor-informed ctDNA mutation data (n=155). The patients were classified according to their tumor-informed ctDNA mutation status and timMRD scores for Plasma B and Plasma C separately. The DFS of each patient was computed from the date of surgery until radiological confirmation of disease relapse. Tick marks indicate patients who were disease-free at data cut-off date. The risk table below the KM plot summarizes the number of patients included per time point. KM survival analysis was performed with log-rank statistics to compare the survival between the two subgroups. Hazard ratio (HR) and corresponding 95% confidence intervals (CI) were computed using univariate Cox proportional-hazards regression model.

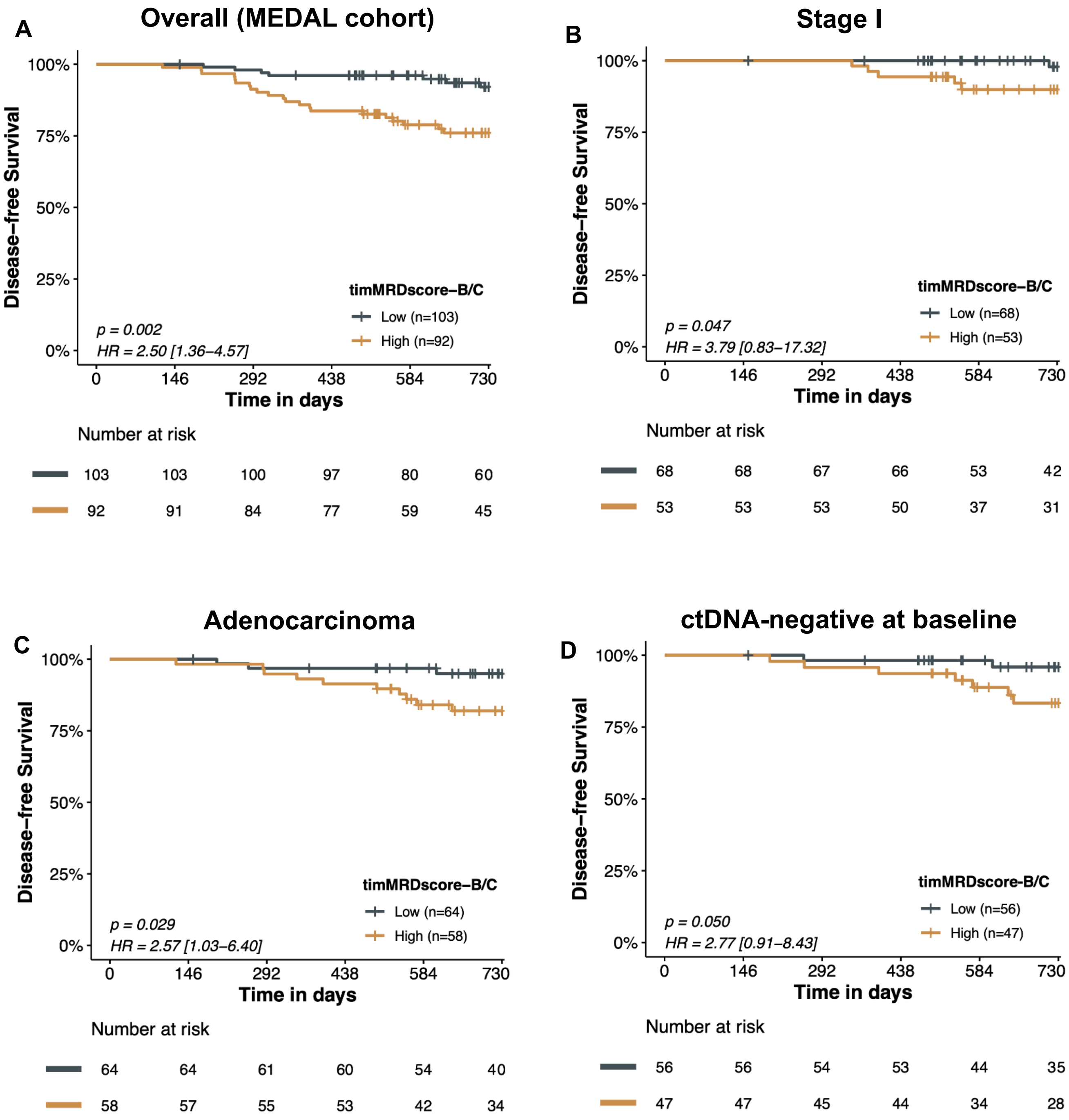

Figure S12. Two-year prognostication using timMRD score evaluated at postoperative follow-up time-points using Plasma B or Plasma C in MEDAL cohort. Kaplan-Meier (KM) analysis comparing the disease-free survival (DFS) (expressed in days) of patients stratified according to timMRD score evaluated for either Plasma B or Plasma C for the MEDAL cohort (**A**) and subgroups with low tumor burden, including stage **I** (n=121) (**B**), adenocarcinoma (n=122) (**C**), and ctDNA negative status at baseline (n=103) (**D**). The risk table below the KM plot summarizes the number of patients included per time point. KM survival analysis was performed with log-rank statistics to compare the survival between the two subgroups. Univariate Cox proportional-hazards regression model was performed to compute the hazard ratio (HR) and corresponding 95% confidence intervals (CI). Statistical significance was defined as P-value <0.05.

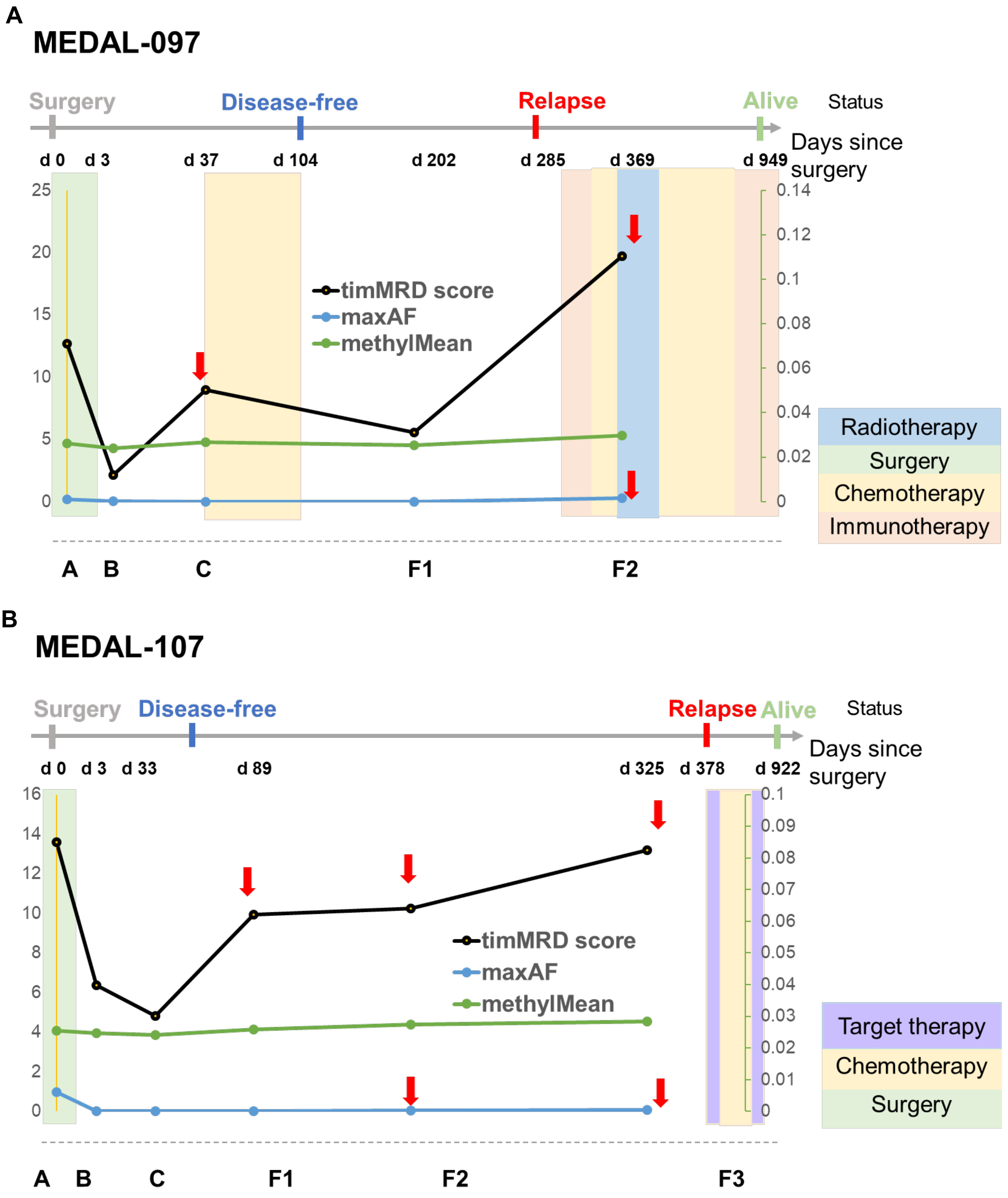

Figure S13. Dynamics of ctDNA mutation and timMRD score in two relapsed patients of the MEDAL cohort. Somatic mutations (blue), MethylMean (green), and timMRD scores (yellow) at baseline (Plasma A), at 3-days post-surgery (Plasma B), at a median of 1-month post-surgery (Plasma C), and at other postoperative follow-up (Plasma F) (more specifically indicated as first (F1), second (F2), and third (F3) when more than 1 postoperative follow-up samples are available), revealing the dynamic patterns that could indicate molecular residual disease. The red arrow indicates the time-point when timMRD score was considered positive. The colored shading corresponds to the treatment received by each patient. **A.** MEDAL-097 was detected with high timMRD score at point C with a lead time of 248 days before radiological confirmation of disease relapse. CtDNA mutation was detected at point B, with a lead time of 282 days before radiological means. **B**. MEDAL-107 was detected with high timMRD score at F1 (89 days post-surgery), with a lead time of 289 days before radiological means, whereas ctDNA mutation was detected at F2 (182 days post-surgery), with a lead time of 196 days.
