# Supplementary material for: Individualized dynamic methylation-based analysis of cell-free DNA in postoperative monitoring of lung cancer
Source: BMC Med. 2023 Jul 14;21:255. doi: 10.1186/s12916-023-02954-z (PMC10349423; doi:10.1186/s12916-023-02954-z)
Supplement: Supplementary file 3 — Additional file 3: Table S1. Baseline clinicopathologic characteristics of the 36 patients in the DYNAMIC cohort. Table S2. Summary of data in related studies on ctDNA-based MRD detection in early-stage lung cancer. Table S3. Analytical performance of timMRD model at various cutoff levels (percentile of chi-square distribution for timMRD scores) and tumor-informed ctDNA mutation status for the 155 patients with tumor-informed ctDNA mutation data at the last follow-up. Of the 155 patients, 11 patients had follow-up data at 120 days before relapse and 52 patients had follow-up data at the same time-point. [file 12916_2023_2954_MOESM3_ESM.docx]

**Supplementary Table S1.** Baseline clinicopathologic characteristics of the 36 patients in the DYNAMIC cohort

| Clinicopathologic features | Disease-free  (n=25); n (%) | Relapsed  (n=11); n (%) | p-value |
| --- | --- | --- | --- |
| Age (median, range) | 62[44-78] | 66[39-75] | 0.30 |
| Sex |  |  |  |
| Female | 11(44.0%) | 5(45.5%) | 1.00 |
| Male | 14(56.0%) | 6(54.5%) |  |
| Smoking history |  |  |  |
| Non-smoker | 14(56.0%) | 9(81.8%) | 0.26 |
| Smoker | 11(44.0%) | 2(18.2%) |  |
| Histology |  |  |  |
| Lung adenocarcinoma | 20(80.0%) | 8(72.7%) | 0.76 |
| Lung squamous cell carcinoma | 4(16.0%) | 3(27.3%) |  |
| Other NSCLC | 1(4.0%) | 0(0%) |  |
| Pathological stage |  |  |  |
| I | 23 (92.0%) | 5 (45.4%) |  |
| II | 1(4.0%) | 2(18.2%) |  |
| IIIA | 1(4.0%) | 3(27.3%) |  |
| IVA | 0(0%) | 1(9.1%) |  |
| Tumor (T) stage |  |  |  |
| 1b | 12(44.0%) | 1(18.1%) | 0.03 |
| 1c | 5(20.0%) | 3(27.3%) |  |
| 2a | 8(36.0%) | 4(27.3%) |  |
| 2b | 0(0%) | 1(9.1%) |  |
| 3 | 0(0%) | 1(9.1%) |  |
| 4 | 0(0%) | 1(9.1%) |  |
| Lymph node (N) stage |  |  |  |
| 0 | 24(92.0%) | 5(54.5%) | 0.001 |
| 1 | 1(4.0%) | 3(27.3%) |  |
| 2 | 0(4.0%) | 3(18.2%) |  |
| Metastasis (M) stage |  |  |  |
| 0 | 25(100%) | 10(90.9%) | 0.31 |
| 1 | 0(0%) | 1(9.1%) |  |
| Visceral pleural involvement | | | |
| With | 7(28.0%) | 3(27.3%) | 0.12 |
| Without | 18(72.0%) | 6(54.5%) |  |
| No data | 0(0%) | 2(18.2%) |  |
| Intravascular tumor thrombus | | | |
| Absent | 21(84.0%) | 3(27.3%) | 0.002 |
| Present | 4(16.0%) | 8(72.7%) |  |
| Surgical method | | | |
| Lobectomy | 23(92.0%) | 10(90.9%) | 1.00 |
| Wedge resection | 2(8.0%) | 1(9.1%) |  |
| Devascularization technique | | | |
| Arterial first | 19(76.0%) | 8(72.7%) | 1.00 |
| Venous first | 4(16.0%) | 2(18.2%) |  |
| No data | 2(8.0%) | 1(9.1%) |  |
| Duration of surgical procedure (median, range) | 135[60~225] | 130[90~300] | 0.21 |
| Tumor diameter, cm (median, range) | 2.3[1.1~3.7] | 2.7[1.9~9.3] | 0.08 |

**Supplementary Table S2.** Summary of data in related studies on ctDNA-based MRD detection in early-stage lung cancer

| Study | N | Stage | Group | ctDNA assay | Sequencing depth | Panel | Lead-time (days) |
| --- | --- | --- | --- | --- | --- | --- | --- |
| TRACERx 2017 ^1^ | 24 | ⅠA-ⅢB | Europe | Natera Signatera (tumor-informed) | 40000x | 18 genes | 70 |
| CAPP-seq 2017 ^2^ | 37 | ⅠB-ⅢB | North America | CAPP⁃seq (tumor-naive) | 10000x | 128 genes | 156 |
| DYNAMIC 2019 ^3^ | 25 | Ⅰ-Ⅲ | East Asia | cSMART (tumor-naive) | 20000x | 9 genes | 165 |

References

1 Abbosh, C. *et al.* Phylogenetic ctDNA analysis depicts early-stage lung cancer evolution. *Nature* **545**, 446-451, doi:10.1038/nature22364 (2017).

2 Chaudhuri, A. A. *et al.* Early Detection of Molecular Residual Disease in Localized Lung Cancer by Circulating Tumor DNA Profiling. *Cancer Discov* **7**, 1394-1403, doi:10.1158/2159-8290.CD-17-0716 (2017).

3 Chen, K. *et al.* Perioperative Dynamic Changes in Circulating Tumor DNA in Patients with Lung Cancer (DYNAMIC). *Clin Cancer Res* **25**, 7058-7067, doi:10.1158/1078-0432.CCR-19-1213 (2019).

**Supplementary Table S3.** Analytical performance of timMRD model at various cutoff levels (percentile of chi-square distribution for timMRD scores) and tumor-informed ctDNA mutation status for the 155 patients with tumor-informed ctDNA mutation data at the last follow-up. Of the 155 patients, 11 patients had follow-up data at 120 days before relapse and 52 patients had follow-up data at the same time-point.

| Assay |  | 98.0^th^ percentile | | 99.0^th^ percentile | | 99.95^th^ percentile | | 99.99^th^ percentile | |
| --- | --- | --- | --- | --- | --- | --- | --- | --- | --- |
|  |  | Negative | Positive | Negative | Positive | Negative | Positive | Negative | Positive |
| timMRD | Disease-free | 35 | 17 | 38 | 14 | 47 | 5 | 51 | 1 |
|  | Relapse | 1 | 10 | 2 | 9 | 5 | 6 | 6 | 5 |
|  | Specificity | 67.3% | | 73.1% | | 90.4%* | | 98.1% | |
|  | Sensitivity | 90.9% | | 81.8% | | 54.5% | | 45.5%^#^ | |
|  | NPV | 97.2% | | 95.0% | | 90.4% | | 89.5% | |
|  | PPV | 37.0% | | 39.1% | | 54.5% | | 83.3% | |
|  | | | | | | | | | |
| Tumor-informed ctDNA mutation | Disease-free | 47 | 5 |  | | | | | |
|  | Relapse | 6 | 5 |  | | | | | |
|  | Specificity | 90.4%* | |  | | | | | |
|  | Sensitivity | 45.5%^#^ | |  | | | | | |
|  | NPV | 88.7% | |  | | | | | |
|  | PPV | 50.0% | |  | | | | | |

Note: The timMRD score cutoff used for the study was 98.0^th^ percentile as described in the Supplementary Methods.

Asterisk (*) denotes the same specificity level between tumor-informed ctDNA mutation status and timMRD-score at 99.95^th^ percentile. This analysis was performed for comparative purposes.

Hash symbol (#) denotes the same sensitivity level between tumor-informed ctDNA mutation status and timMRD-score at 99.99^th^ percentile. This analysis was performed for comparative purposes.

Abbreviations: NPV, negative predictive value; PPV, positive predictive value
